# Molecular phylogenetics of swimming crabs (Portunoidea Rafinesque, 1815) supports a revised family-level classification and suggests a single derived origin of symbiotic taxa

**DOI:** 10.7717/peerj.4260

**Published:** 2018-01-23

**Authors:** Nathaniel Evans

**Affiliations:** 1 Department of Invertebrate Zoology, National Museum of Natural History, Smithsonian Institution, Washington, DC, USA; 2 Division of Invertebrate Zoology, Florida Museum of Natural History, University of Florida, Gainesville, FL, USA

**Keywords:** Evolution, Taxonomy, Phylogeny, Portunidae, Portunoidea, Symbiosis, Decapoda, Brachyura, Systematics, Swimming crab

## Abstract

Portunoidea is a diverse lineage of ecologically and economically important marine crabs comprising 8 families and 14 subfamilies. Closely related portunid subfamilies Caphyrinae and Thalamitinae constitute some of this group’s greatest morphological and taxonomic diversity, and are the only known lineages to include symbiotic taxa. Emergence of symbiosis in decapods remains poorly studied and portunoid crabs provide an interesting, but often overlooked example. Yet the paucity of molecular phylogenetic data available for Portunoidea makes it challenging to investigate the evolution and systematics of the group. Phylogenetic analyses, though limited, suggest that many putative portunoid taxa are para- or polyphyletic. Here I augment existing molecular data—significantly increasing taxon sampling of Caphyrinae, Thalamitinae, and several disparate portunoid lineages—to investigate the phylogenetic origin of symbiosis within Portunoidea and reevaluate higher- and lower-level portunoid classifications. Phylogenetic analyses were carried out on sequences of H3, 28S rRNA, 16S rRNA, and CO1 for up to 168 portunoid taxa; this included, for the first time, molecular data from the genera *Atoportunus*, *Brusinia*, *Caphyra*, *Coelocarcinus*, *Gonioinfradens*, *Raymanninus*, and *Thalamonyx*. Results support the placement of all symbiotic taxa (*Caphyra*, *Lissocarcinus*, and two *Thalamita*) in a single clade derived within the thalamitine genus *Thalamita*. Caphyrina Paulson, 1875, nom. trans. is recognized here as a subtribe within the subfamily Thalamitinae. Results also support the following taxonomic actions: *Cronius* is reclassified as a thalamitine genus; *Thalamonyx* is reestablished as a valid genus; *Goniosupradens* is raised to the generic rank; and three new genera (*Zygita* gen. nov., *Thranita* gen. nov., and *Trierarchus* gen. nov.) are described to accommodate some *Thalamita* s.l. taxa rendered paraphyletic by Caphyrina. A new diagnosis of Thalamitinae is provided. Results also support a more conservative classification of Portunoidea comprising three instead of eight extant families: Geryonidae (Geryonidae + Ovalipidae; new diagnosis provided), Carcinidae (Carcinidae + Pirimelidae + Polybiidae + Thiidae + *Coelocarcinus*; new diagnosis provided) and Portunidae. Finally, 16s rRNA data suggests family Brusiniidae might not be a portunoid lineage.

## Introduction

**Figure 1:**
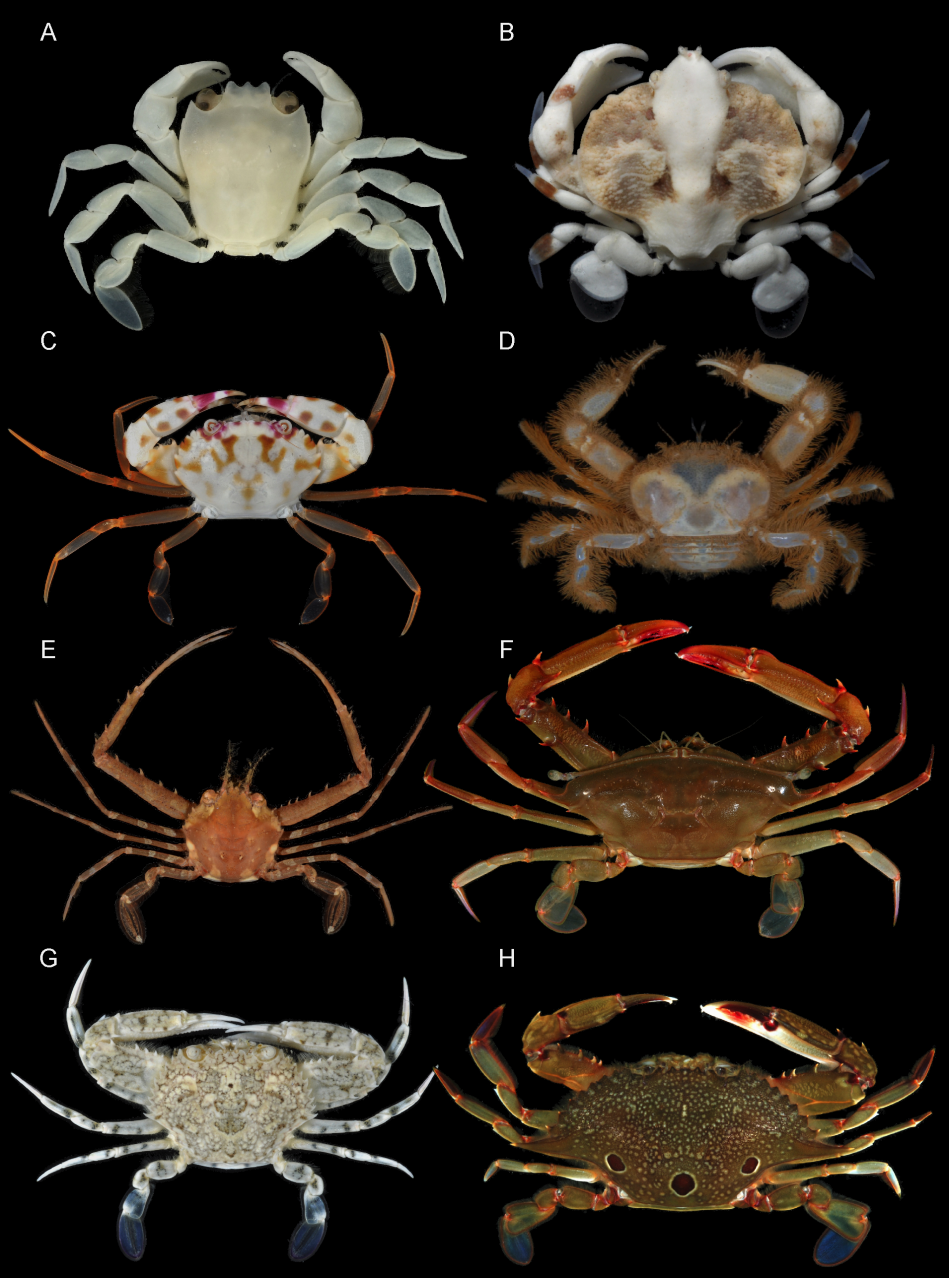
Representatives of various Portunoidea taxa included in this study. (A) *Brusinia profunda* (USNM 277519; New Caledonia; preserved color); (B) *Coelocarcinus foliatus* (UF 40176; Guam); (C) *Carupa tenuipes* (UF 39918; Palau); (D) *Libystes* (UF 23926; Moorea Is.); (E) *Lupocyclus* cf. *philippinensis* (UF 41639; Luzon Is.); (F) *Podophthalmus vigil* (UF 24543; Moorea Is.); (G) *Portunus (Cycloachelous) granulatus* (UF 40021; Guam); (H) *Portunus (Portunus) sanguinolentus* (UF 24538; Moorea Is.). Photographs (A–C, G) by Nathaniel Evans; photographs (D–F, H) by Gustav Paulay.

**Figure 2:**
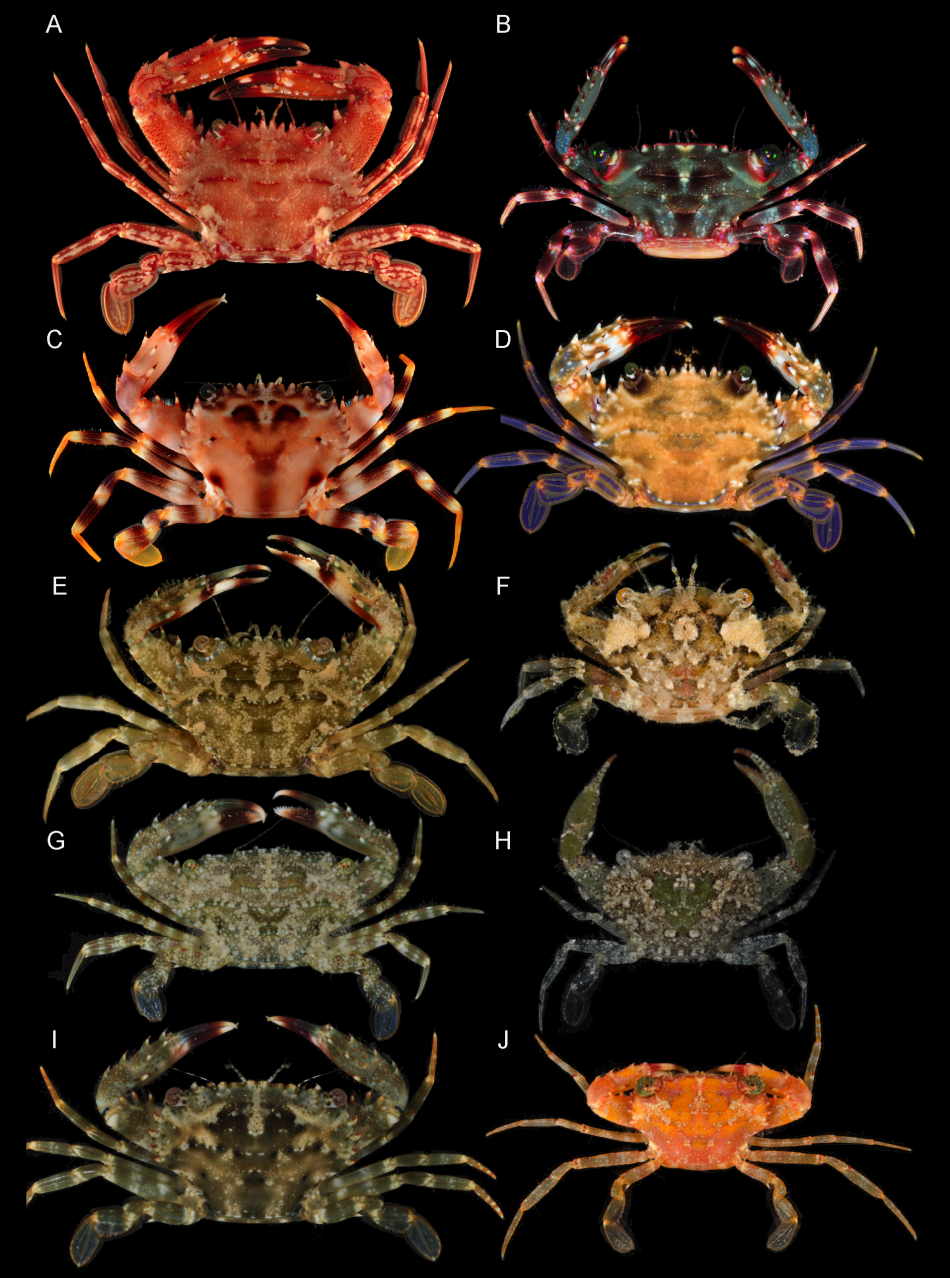
Representative non-symbiotic Thalamitinae species. (A) *Cronius ruber* (UF 35672; Florida); (B) *Thalamitoides spinigera* (UF 36697; Farasan Banks); (C) *Gonioinfradens paucidentatus* (UF 37141; Red Sea); (D) *Goniosupradens acutifrons* (UF 7114; Okinawa); (E) *Charybdis orientalis* (UF 41638; Luzon Is.); (F) *Thalamonyx gracilipes* (UF 42972; Mindoro Is.); (G) *Thalamita admete* (UF 40031; Guam); (H) *Thalamita chaptalii* (UF 39917; Palau); (I) *Thranita coeruleipes,* comb. nov. (UF 40078; Guam); (J) *Thalamita cf. philippinensis* (UF 43302; Mindoro Is.). Photographs (A, G–I) by Nathaniel Evans; photographs (B–F, J) by Gustav Paulay.

**Figure 3:**
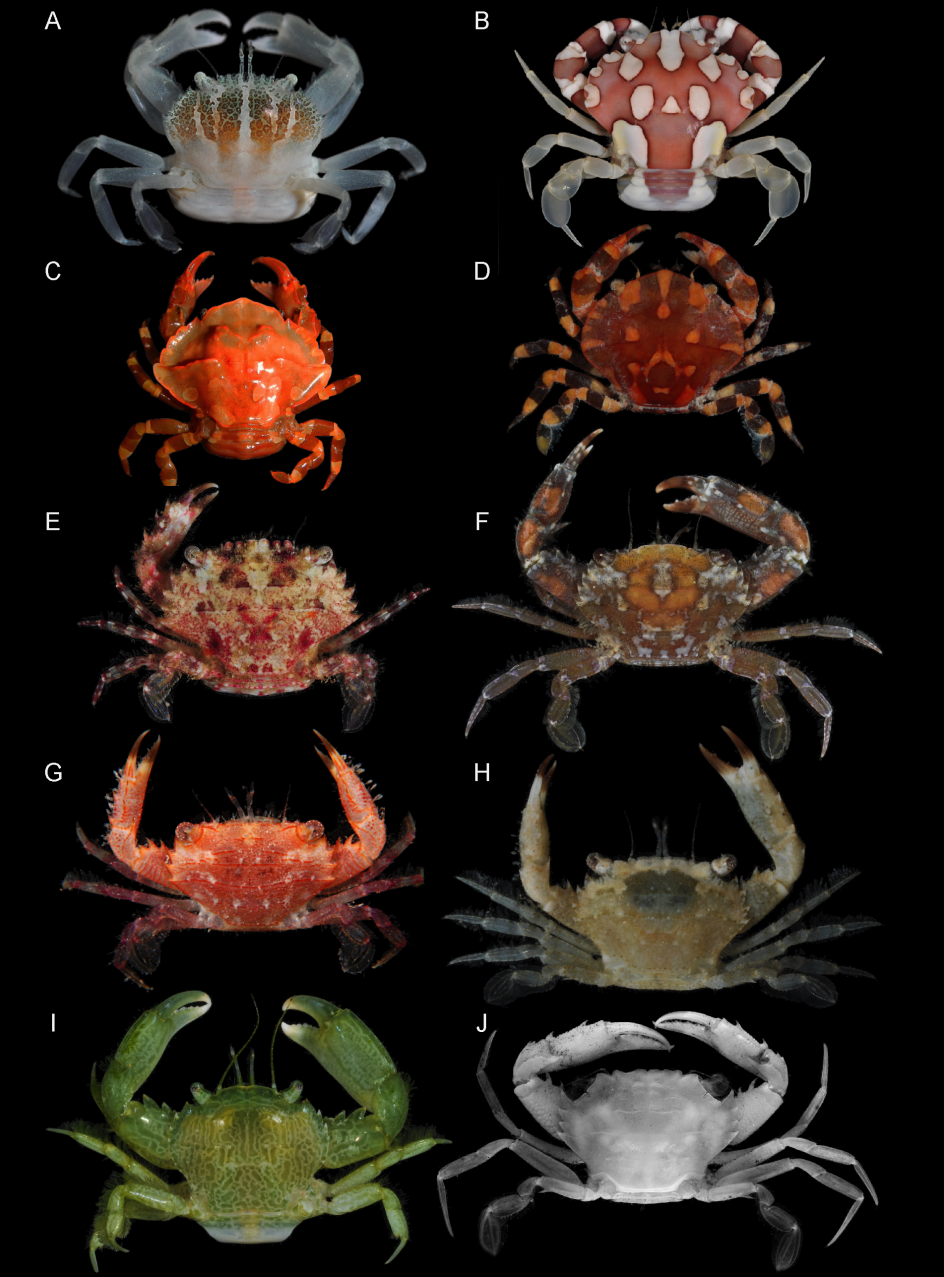
Representative putative symbiotic Thalamitinae species. (A) *Caphyra loevis* (UF 39060); (B) *Lissocarcinus* cf. *laevis* (UF 39136; New Caledonia); (C) *Lissocarcinus holothuricola* (UF 30182; Marquesas); (D) *Lissocarcinus orbicularis* (UF 23972; Moorea); (E) *Zygita murinae*, comb. nov. (UF 36721; Farasan Banks); (F) *Trierarchus woodmasoni*, comb. nov. (UF 40079; Guam); (G) *Trierarchus* cf. *cooperi* sp. A, comb. nov. (UF 16023; Moorea Is.); (H) *Trierarchus* cf. *cooperi* sp. B, comb. nov. (UF 40100; Guam); (I) *Trierarchus rotundifrons,* comb. nov. (UF 40067; Guam); (J) *Trierarchus squamosus*, comb. nov. (USNM 102963; Bikini Atoll; preserved specimen, grayscale, left frontal margin damaged). Photographs (A–C, F, H–J) by Nathaniel Evans; photographs (D, E, G) by Gustav Paulay.

**Figure 4:**
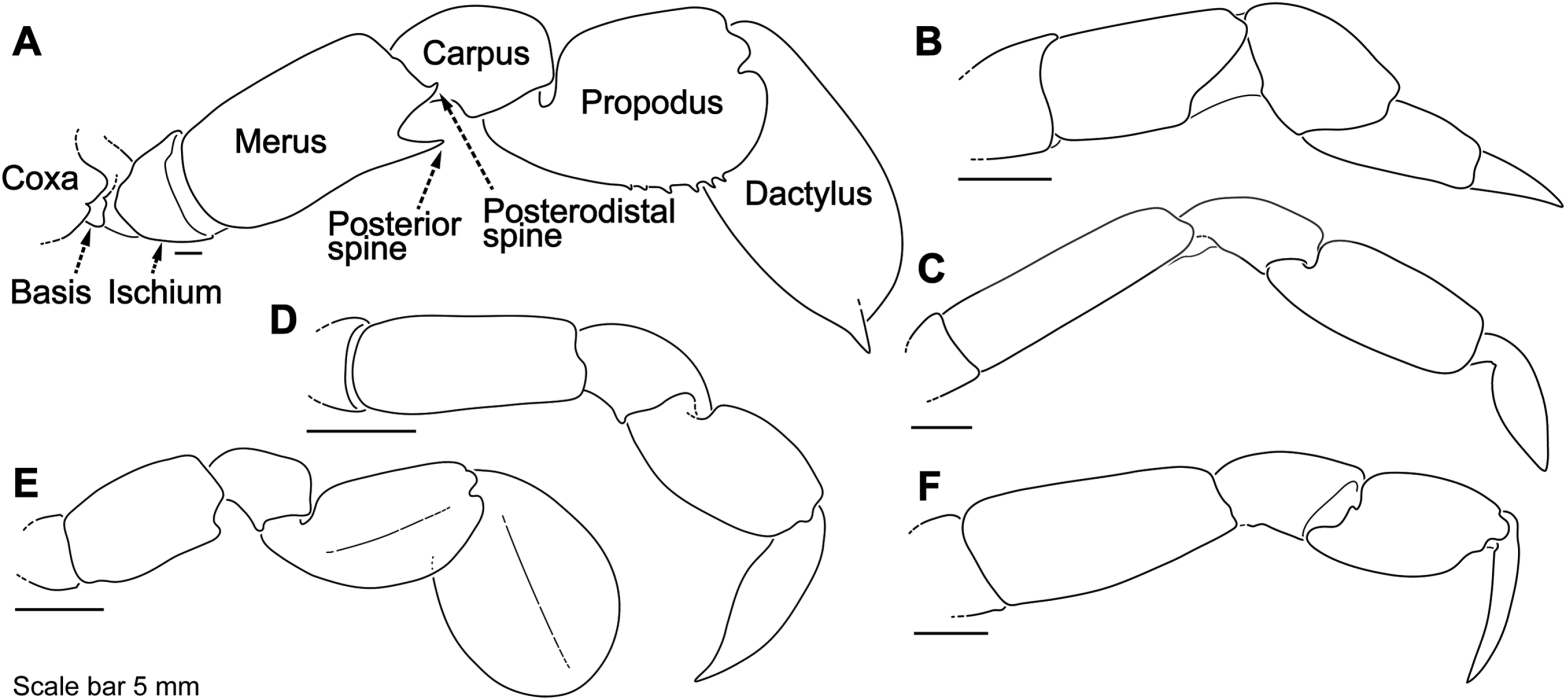
Representative portunid natatory leg morphology and divergent, symbiotic caphyrine forms. Typical portunid P5 morphology and terminology: (A) *Thranita* cf. *rubridens* (UF 43834). Typical symbiotic caphyrine P5 morphology: (B) *Caphyra* cf. *fulva* (UF 11748; host xeniid soft coral); (C) *Caphyra loevis* (UF 38881; host xeniid soft coral); (D) *Lissocarcinus holothuricola* (UF 30302; host holothurians); (E) *Lissocarcinus laevis* (UF 41571; host cerianthids and actinodendronid anemones); (F) *Caphyra rotundifrons* (=*Trierachus rotundifrons*, comb. nov., UF 40067A; host *Chlorodesmis* algae).

The superfamily Portunoidea Rafinesque, 1815 (455 spp.; De Grave et al., 2009) is a diverse clade of marine crabs that includes commercially important species, significant invasives (Brockerhoff & McLay, 2011) and several ecologically divergent lineages that radiated across tropical, temperate and deep-ocean habitats (e.g., Figs. 1 and 2). Collectively referred to as “swimming crabs,” members of this clade are known for being aggressive opportunistic omnivores that are agile and well adapted to swimming (Hartnoll, 1971; Hazlett, 1971; Spiridonov, Neretina & Schepetov, 2014; Williams, 1981). Morphologically, portunoid crabs are characterized by having a broad, compressed, laterally streamlined carapace and paddle-shaped posterior “natatory” legs (Hartnoll, 1971). Yet this clade also includes several atypical lineages that are morphologically and ecologically divergent. Among these, members of the tropical Indo-Pacific subfamily Caphyrinae Paulson, 1875 (28 spp.) have evolved symbiotic relationships with algae, anemones, echinoderms, and soft corals (Caulier et al., 2013; Hay et al., 1989; Spiridonov, 1999; Stephenson & Rees, 1968). Relative to most portunoids, members of this group are smaller, less streamlined and exhibit highly modified “natatory” legs adapted for grasping onto or burying beneath their hosts (Figs. 3A–3D, 3I and 4B–4F). Additional adaptations to symbiosis found in these crabs include cryptic coloration (Ayotte, 2005), attraction to host chemical defense compounds (Caulier et al., 2013; Hay et al., 1989), consumption of host tissue (Caulier et al., 2014; Hay et al., 1989; Steudler, Schmitz & Ciereszko, 1977), and social monogamy (Caulier et al., 2012; for significance see Baeza & Thiel, 2007). Despite its novelty among portunoid crabs, the nature of symbiosis in Caphyrinae remains poorly studied and underreported (Baeza, 2015; Castro, 2015). Unlike Caphyrinae, most well-studied symbiotic crustaceans fall within clades that are species-rich and dominated by or exclusively composed of symbiotic taxa (Baeza, 2015). This has led some to hypothesize that the emergence of symbiosis in crustaceans promotes large evolutionary radiations (Baeza, 2015). However, this hypothesis remains to be tested, requiring phylogenetic analyses of multiple clades with symbiotic and free-living lineages.

Recently, Evans & McKeon (2016) provided compelling evidence that some species of the portunid genus *Thalamita* also exhibit symbiotic relationships (with soft coral). It has long been suggested that Caphyrinae shares a close, even derived relationship with *Thalamita* and other taxa in the diverse portunid subfamily Thalamitinae Paulson, 1875 (162 spp.; e.g., see Stephenson & Campbell, 1960). Thalamitinae radiated across the same Indo-Pacific habitats where Caphyrinae and their reef-associated host taxa are found. Consequently, Caphyrinae and Thalamitinae provide an interesting group to investigate the evolution of symbiosis in decapod crustaceans. Unfortunately, like much of Portunoidea, little phylogenetic work has been done on Thalamitinae or Caphyrinae.

The original aim of this study was to investigate the molecular phylogenetic relationships within and between Thalamitinae and Caphyrinae, providing important context for understanding the evolution of symbiosis within portunids. However, preliminary analyses revealed that inclusion of the non-symbiotic Caphyrinae genus *Coelocarcinus* required analyses be expanded to include the entire superfamily Portunoidea. Consequently, this study compiles and augments the best available molecular data for all of Portunoidea (as of January 2017). Given this broader scope, here I also reevaluate family classifications within the superfamily Portunoidea and subfamily classifications within Portunidae. Finally, for Thalamitinae and Caphyrinae, where taxon sampling is now the densest of any portunoid clade, generic level classifications are also reevaluated and new genera and morphological diagnoses proposed where appropriate.

### A brief review of portunoid systematics

Considerable systematic work was carried out on Portunoidea during the 19th and 20th centuries, often in conjunction with work on the morphologically similar Cancroidea (reviewed in Davie, Guinot & Ng, 2015a; Karasawa, Schweitzer & Feldmann, 2008; Schubart & Reuschel, 2009). Toward the end of this period W. Stephenson revised and largely stabilized portunoid classification (Stephenson, 1972). However, morpho-taxonomic work has continued for the group, sometimes revealing surprisingly unique new lineages (e.g., *Atoportunus* Ng & Takeda, 2003). In recent years genetic data has increasingly been combined with morphology to resolve species complexes (Keenan, Davie & Mann, 1998; Lai, Ng & Davie, 2010; Robles et al., 2007), but neither molecular nor morphological phylogenetic analyses have been widely applied to the group.

To date, only three studies have conducted higher-level molecular phylogenetic analyses of Portunoidea, using 16S rRNA or combinations of CO1, H3, 16S and 28S rRNA for up to 43 portunoid taxa (Mantelatto et al., 2009; Schubart & Reuschel, 2009; Spiridonov, Neretina & Schepetov, 2014). Of these studies, the latter two are the only to include a caphyrine species (*Lissocarcinus orbicularis*), which was recovered falling sister to, or derived within Thalamitinae (comprised of one and six thalamitine taxa, respectively). Though these studies have significantly improved our understanding of portunoid systematics, synthesis of this work is complicated by a lack of overlap in both taxa and molecular data sampled.

**Figure 5:**
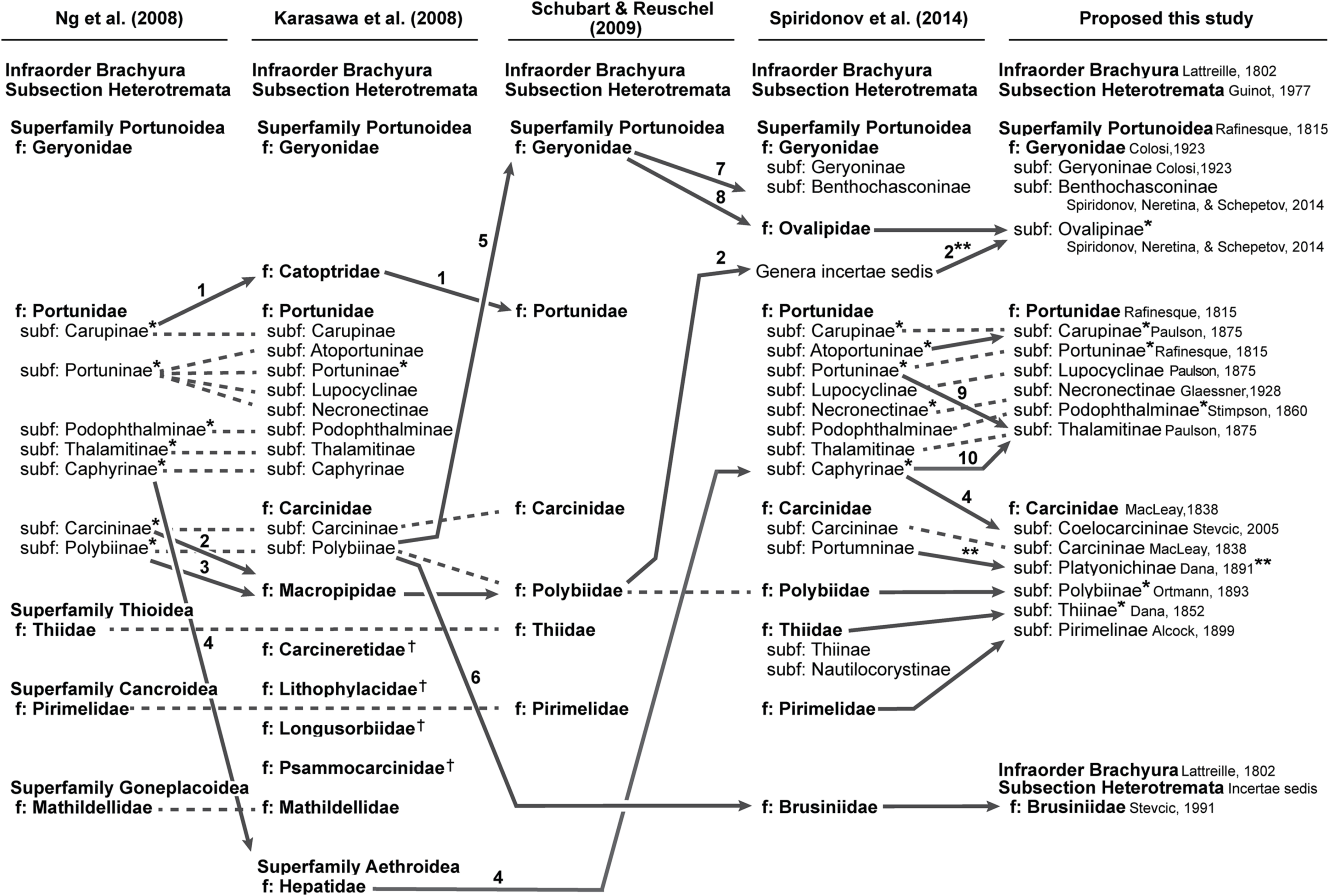
Summary of major recent changes to Portunoidea familial and subfamilial classification and a new proposed scheme. Dashed and arrowed lines trace recognized taxa between studies. Solid arrowed lines highlight notable changes, with numbers indicating the movement of specific genera: 1, *Catoptrus* and *Libystes*; 2, *Echinolatus* and *Nectocarcinus*; 3, *Bathynectes*, *Macropipus*, *Necora*, *Parathranites* and *Raymanninus*; 4, *Coelocarcinus*; 5, *Benthochchascon* and *Ovalipes*; 6, *Brusinia*; 7, *Benthochchascon*; 8, *Ovalipes*; 9, *Cronius*; 10, *Caphyra* and *Lissocarcinus*. Single asterisk*, corresponding study considers subfamily composition and status uncertain given morphological or phylogenetic results, or lack there of; double asterisks**, change made following Davie, Guinot & Ng (2015b); †, extinct family. Figure modeled after Fig. 8 in Spiridonov, Neretina & Schepetov (2014).

In addition to molecular work, only the generic level morphological cladistic analyses of Portunoidea by Karasawa, Schweitzer & Feldmann (2008) have significantly contributed to our understanding of higher-level phylogenetic relationships within the clade. None of this work analyzed more than approximately 40 of the 455 extant portunoid taxa. Nevertheless, beginning with Ng, Guinot & Davie (2008) four different schemes have been proposed for the familial and subfamilial classification of Portunoidea (Fig. 5). While additional revisions will likely be needed, here I propose a new, more conservative classification scheme for extant portunoids based on more comprehensive molecular phylogenetic analyses of the superfamily.

## Materials and Methods

### Voucher material and taxonomic identifications

Sequence data generated for this study was derived from 137 vouchered specimens listed in Table 1 and Table S1 from the following collections: the National Museum of Marine Biology and Aquarium, Taiwan (NMMBCD); the Florida Museum of Natural History, University of Florida, Gainesville, Florida, USA (UF); the National Museum of Natural History, Smithsonian Institution, Washington, DC, USA (USNM); the Zoological Reference Collection of the Lee Kong Chian Natural History Museum, National University of Singapore, Singapore (ZRC). Additional information regarding UF and USNM vouchers can be obtained by searching digital collection records (http://specifyportal.flmnh.ufl.edu/iz/ and https://collections.nmnh.si.edu/search/iz/) or through the iDigBio portal (www.idigbio.org/portal/search). Morphological work was conducted using these and other specimens in UF and USNM holdings. Species identifications were made using taxonomic literature (Edmondson, 1954; Stephenson, 1972; Stephenson & Hudson, 1957; Wee & Ng, 1995) and with reference to material (including types) previously identified by M.J. Rathbun, W. Stephenson, or V. Spiridonov. Identification and taxon sampling was also aided through analyses of a large unpublished data set of CO1 DNA barcode sequences generated from over 1,000 USNM and UF portunoid specimens. Inclusion of all DNA barcode data is beyond the scope of this study but is forthcoming in several investigations led by C.P. Meyer, G. Paulay or N. Evans. The classification scheme of Ng, Guinot & Davie (2008) was primarily followed here including, for the sake of clarity, the *Portunus* subgeneric classification scheme. However, some modifications were made to be consistent with Spiridonov, Neretina & Schepetov (2014). Specifically, *Cycloachelous* was treated as a valid subgenus and *Lupocycloporus* a valid genus. Lineage specific taxa counts were taken from Davie, Guinot & Ng (2015b), De Grave et al. (2009) and Spiridonov, Neretina & Schepetov (2014) and typically do not reflect changes made after these works. Finally, following clarification by V. Spiridonov (2017, personal communication) the authorship of Caphyrinae, Carupinae, Lupocylinae, and Thalamitinae are attributed to Paulson (1875). This avoids the widely used misspelling Paul’son, which resulted from an improper English translation of Paulson (1875) from Cyrillics. Original translations of the author’s name in Latin were Paulson and Paulsohn, but never Paul’son.

**Table 1:** Taxon sampling, GenBank accession numbers, and operational taxonomic unit (OTU) composition of sequence data used for phylogenetic analyses

Taxon	16S rRNA	CO1	H3	28S rRNA	Notes	Voucher ID
**Cancroidea: Cancridae:**						
*Cancer pagurus* Linnaeus, 1758	FM207653	*JQ306000	**DQ079668	**DQ079781	A, B	SMF32764/*MB89000194/**BYU-KC2158
**Carpilioidea: Carpiliidae:**						
*Carpilius convexus* (Forskal, 1775)	FM208748	*JX398091	*JX398111	*JX398073	A	SMF32771/*ZMMUMa3438
**Corystoidea: Corystidae:**						
*Corystes cassivelaunus* (Pennant, 1777)	FM208781	*JQ306006	FM208801	NA	A	SMF32770/*MB89000203
**Eriphioidea: Menippidae:**						
*Menippe rumphii* (Fabricius, 1798)	HM637976	HM638051	HM596626	NA		ZRC2003.211
**Parthenopoidea: Parthenopidae:**						
*Daldorfia horrida* (Linnaeus, 1758)	GQ249177	*HM638031	GQ249174	NA		ZRC2003.0651
**Xanthoidea: Xanthidae:**						
*Etisus utilis* Jacquinot, 1853	HM798456	HM750981	*JX398108	NA		ZRC2002.0586/*NA
**Portunoidea: Carcinidae: Carcininae**						
*Carcinus maenas* (Linnaeus, 1758)	FM208763	*FJ581597	FM208811	**DQ079798	A, B	SMF32757/*NA/**BYU-KACmapu
**Portunoidea: Carcinidae: Coelocarcininae**						
*Coelocarcinus* aff. *foliatus*	**KT365545**	NA	NA	NA	A	UF27553
*Coelocarcinus foliatus* Edmondson, 1930	**KT365601**	**KT365724**	**KT425058**	NA		UF40056
**Portunoidea: Carcinidae: Pirimelinae**						
*Pirimela denticulata* (Montagu, 1808)	FM208783	NA	FM208808	NA	A	SMF32767
*Sirpus zariquieyi* Gordon, 1953	FM208784	NA	FM208809	NA	A	SMF32768
**Portunoidea: Carcinidae: Platyonichinae**						
*Portumnus latipes* (Pennant, 1777)	FM208764	NA	FM208812	NA	A	SMF32758
**Portunoidea: Carcinidae: Polybiinae**						
*Liocarcinus corrugatus (Pennant, 1777)*	GQ268542	GQ268536	*FM208820	NA		NA/*SMF32760
*Liocarcinus depurator (Linnaeus, 1758)*	FM208767	*FJ174948	*FJ174852	*FJ036939	A	MNHNB31439/*NA
*Liocarcinus holsatus (Fabricius, 1798)*	FM208766	*GQ268538	FM208817	NA	A	SMF32750/*NA
*Liocarcinus maculatus (Risso, 1827)*	FJ174892	FJ174949	FJ174853	FJ036940		NA
*Liocarcinus marmoreus (Leach, 1814)*	GQ268547	GQ268535	NA	NA		NA
*Liocarcinus navigator (Herbst, 1794)*	GQ268541	GQ268537	*FM208821	NA		NA/*SMF32775
*Liocarcinus vernalis (Risso, 1816)*	FM208768	*JX123455	NA	NA	A	SMF32761/*CCDB-1739
*Bathynectes longispina* (Risso, 1816)	**KT365526**	*KT365693	NA	**KT365627**	A, B	UF9383/*UF15140
*Bathynectes maravigna* (Prestandrea, 1839)	FM208770	*JQ305966	FM208814	NA	A	MNHNB31441/*NA
*Macropipus tuberculatus* (Roux, 1830)	FM208769	*GQ268530	FM208815	NA	A	MNHNB31440/*NA
*Necora puber* (Linnaeus, 1767)	FM208771	*FJ755619	FM208813	**DQ079800	A, B	SMF32749/*NA/**BYU-KAC2161
*Parathranites orientalis* (Miers, 1886)	KJ132616	NA	KJ133173	NA		NTOUB00090
*“Polybius” henslowii* Leach, 1820	FM208765	*JQ306041	FM208816	NA	A	SMF32759/*MB89000200
**Portunoidea: Carcinidae: Thiinae**						
*Thia scutellata* (Fabricius, 1793)	FM208782	NA	FM208810	NA	A	SMF32769
**Portunoidea: Geryonidae: Benthochasconinae**						
*Benthochascon hemingi* Alcock & &erson, 1899	FM208772	*HM750955	FM208826	NA	A	ZRC2000.102
**Portunoidea: Geryonidae: Geryoninae**						
*Chaceon granulatus* (Sakai, 1978)	FM208775	*AB769383	FM208827	NA	A	SMF32762/*NA
*Geryon longipes* A. Milne-Edwards, 1882	FM208776	*JQ305902	FM208828	NA	A	SMF32747/*MB89000638
*Raymanninus schmitti* (Rathbun, 1931)	**KT365560**	NA	NA	**KT365656**	A, B	UF9676
**Portunoidea: Geryonidae: Ovalipinae**						
*Ovalipes iridescens* (Miers, 1886)	FM208774	NA	FM208825	NA	A	ZRC1995.855
*Ovalipes punctatus* (De Haan, 1833)	KJ132597	*KF906404	KJ133154	NA		NTOUB00011/*NA
*Ovalipes stephensoni* Williams, 1976/*O.floridanus Hay & Shore, 1918	DQ388050	NA	NA	*KT365648	B	ULLZ5678/*UF28577
*Ovalipes trimaculatus* (De Haan, 1833)	FM208773	*JN315648	FM208823	NA	A	MNHNB19785/*NA
**Portunoidea: Portunidae: Carupinae**						
*Atoportunus gustavi* Ng & Takeda, 2003	**KT365590**	**KT365692**	NA	NA		UF1266
*Carupa ohashii* Takeda, 1993	FM208759	NA	FM208790	NA	A	SMF32756
*Carupa tenuipes* (var. A) Dana, 1852	FM208758	*KT365703	FM208789	NA	A	MNHNB31436/*UF16185
*Carupa tenuipes* (var. B) Dana, 1852	**KT365533**	**KT365704**	NA	NA	A	UF15565
*Catoptrus* aff. *nitidus*	**KT365534**	**KT365706**	NA	NA	A	UF18451
*Catoptrus nitidus* A. Milne-Edwards, 1870/*C. aff. *nitidus*	FM208755	*KT365705	NA	NA	A	MNHNB31435/*UF1024
Laleonectes cf. nipponensis/*L. nipponensis (Sakai, 1938)	**KT365548**	**KT365727**	*FM208792	NA	A	UF7342/*MNHNB31434
*Libystes edwardsii* Alcock, 1899	FM208761	NA	NA	NA	A	MNHNB31437
*Libystes nitidus* A. Milne-Edwards, 1867	FM208762	*KT365728	NA	NA	A	MNHNB31438/*UF12587
*Richerellus moosai* Crosnier, 2003	FM208756	NA	FM208788	NA	A	MNHNB22838 (paratype)
**Portunoidea: Portunidae: Lupocyclinae**						
*Lupocycloporus gracilimanus* (Stimpson, 1858)	AM410523	*JX398092	*JX398124	*JX398076		NA/*ZMMUMa3381
*Lupocyclus philippinensis* Semper, 1880	FJ152156	NA	*JX398119	*JX398077		NA/*ZMMUMa3443
*Lupocyclus quinquedentatus* Rathbun, 1906	**KT365603**	**KT365734**	NA	**KT365647**	B	UF10568
*Lupocyclus rotundatus* Adams & White, 1849	NA	NA	JX398110	JX398075	C	ZMMUMa3441
**Portunoidea: Portunidae: Necronectinae**						
*Scylla olivacea* (Herbst, 1796)	FJ827760	FJ827760	NA	NA	A	NA
*Scylla paramamosain* Estampador, 1949	FJ827761	FJ827761	NA	NA	A	NA
*Scylla serrata* (Forskal, 1775)	FJ827758	FJ827758	*FM208793	NA	A	NA/*MZUF3657
*Scylla tranquebarica* (Fabricius, 1798)	FJ827759	FJ827759	NA	NA	A	NA
**Portunoidea: Portunidae: Podophthalminae**						
*Euphylax robustus* A. Milne-Edwards, 1874	FJ152153	NA	NA	NA		CCDB-1122
*Podophthalmus nacreus* Alcock, 1899	NA	JX398093	NA	JX398078	C	ZMMUMa3440
*Podophthalmus vigil* (Fabricius, 1798)	**KT365553**	**KT365735**	*FM208787	NA	A	UF18116/*ZRCY4821
**Portunoidea: Portunidae: Portuninae**						
*Arenaeus cribrarius* (Lamarck, 1818)	FM208749	*JX123439	FM208799	NA	A	SMF32753/*CCDB-3182
*Arenaeus mexicanus* (Gerstaecker, 1856)	JX123470	JX123446	NA	NA		MZUCR2430-4
*Callinectes marginatus* (A. Milne-Edwards, 1861)	**KT365527**	**KT365694**	NA	NA	A	UF11403
*Callinectes ornatus* Ordway, 1863	**KT365528**	NA	NA	**KT365628**	A, B	UF19804
*Callinectes sapidus* Rathbun, 1896	AY363392	AY363392	*FM208798	**AY739194	A, B	NA/*ULLZ3895/**NA
*Lupella forceps* (Fabricius, 1793)	FJ152155	NA	NA	NA		USNM284565
*Portunus (Achelous) asper* (A. Milne-Edwards, 1861)	FJ152158	NA	NA	NA		CCDB1738
*Portunus (Achelous) depressifrons* (Stimpson, 1859)	DQ388064	*KT365738	NA	NA		ULLZ4442/*UF26120
*Portunus (Achelous) floridanus* Rathbun, 1930	DQ388058	NA	NA	NA		ULLZ4695
*Portunus (Achelous) gibbesii* (Stimpson, 1859)	DQ388057	*KT365739	NA	**KT365650	B	ULLZ4565/*UF1134/**UF19561
*Portunus (Achelous) ordwayi* (Stimpson, 1860)	FM208751	*KT365689	FM208794	NA	A	SMF31988/*UF6426
*Portunus (Achelous) rufiremus* Holthuis, 1959	DQ388063	NA	NA	NA		USNM151568
*Portunus (Achelous) sebae* (H. Milne Edwards, 1834)	DQ388067	NA	NA	NA		ULLZ4527
*Portunus (Achelous) spinicarpus* (Stimpson, 1871)	DQ388061	*KT365746	NA	NA		ULLZ4618/*UF3969
*Portunus (Achelous) spinimanus* Latreille, 1819	**KT365558**	*KT365690	NA	**KT365654**	A, B	UF28417/*UF6692
*Portunus (Achelous) tumidulus* Stimpson, 1871	**KT365589**	**KT365691**	NA	NA		UF32157
*Portunus (Cycloachelous) granulatus* (H. Milne Edwards,1834)	**KT365605**	**KT365740**	NA	**KT365651**	B	UF4169
*Portunus (Cycloachelous) orbitosinus* (Rathbun, 1911)	NA	JX398097	JX398115	JX398082	C	ZMMUMa3378
*Portunus (Monomia) argentatus* (A. Milne-Edwards, 1861)	NA	JX398096	JX398107	JX398081	C	ZMMUMa3365
*Portunus (Monomia) gladiator* Fabricius, 1798	NA	JX398095	JX398113	JX398080	C	ZMMUMa3366
*Portunus (Monomia) petreus* (Alcock, 1899)	**KT365606**	**KT365743**	NA	NA		UF188
*Portunus (Monomia) pseudoargentatus* Stephenson, 1961	NA	JX398094	JX398121	JX398079	C	ZMMUMa3368
*Portunus (Portunus) anceps* (Saussure, 1858)	**KT365604**	**KT365736**	NA	NA		UF32492
*Portunus (Portunus) hastatus* (Linnaeus, 1767)	FM208780	NA	FM208796	NA		SMF31989
*Portunus (Portunus) inaequalis* (Miers, 1881)	FM208752	NA	FM208795	NA	A	SMF32754
*Portunus (Portunus) pelagicus* (Linnaeus, 1758)	FM208750	*JX398106	*JX398116	*JX398074	A	CSIRO uncatalogued/*NA
*Portunus (Portunus) sanguinolentus hawaiiensis* Stephenson, 1968	**KT365557**	**KT365744**	NA	**KT365653**	A, B	UF8949
*Portunus (Portunus) sayi* (Gibbes, 1850)	**KT365607**	**KT365745**	NA	NA		UF26156
*Portunus (Portunus) trituberculatus* (Miers, 1876)	AB093006	AB093006	*FM208829	NA	A	NA/*NA
*Portunus (Portunus) ventralis* (A. Milne-Edwards, 1879)	**KT365559**	**KT365747**	NA	**KT365655**	A, B	UF32351
*Portunus (Xiphonectes) arabicus* (Nobili, 1905)	**KT365554**	**KT365737**	NA	**KT365649**	A, B	UF7735
*Portunus (Xiphonectes) hastatoides* Fabricius, 1798	NA	JX398098	NA	JX398083	C	ZMMUMA3392
*Portunus (Xiphonectes)* aff. *longispinosus*	**KT365555**	**KT365741**	NA	**KT365652**	A, B	UF10477
*Portunus (Xiphonectes) longispinosus* (Dana, 1852)	**KT365556**	**KT365742**	NA	NA	A	UF187
*Portunus (Xiphonectes) tenuipes* (De Haan, 1835)	NA	JX398099	NA	JX398087	C	NA
**Portunoidea: Portunidae: Thalamitinae**						
*Caphyra bedoti* (Zehntner, 1894)	**KT365591**	**KT365695**	**KT425019**	NA		NMMBCD 4091
*Caphyra c*f. *fulva*	**KT365529**	**KT365696**	**KT424990**	**KT365629**	A, B	UF11748
*Caphyra loevis* (A. Milne-Edwards, 1869)	**KT365592**	**KT365697**	**KT425009**	NA		NMMBCD 4090
*Caphyra tridens* Richters, 1880	**KT365532**	**KT365701**	**KT425003**	**KT365632**	A, B	UF15907
*Caphyra yookadai* Sakai, 1933	**KT365593**	**KT365702**	**KT424993**	NA		NMMBCD 4089
*Caphyra* sp. A	**KT365531**	**KT365699**	NA	NA	A	UF5061-A
*Caphyra* sp. B	NA	**KT365700**	**KT425046**	**KT365631**	B, C	UF14454
*Charybdis acuta* (A. Milne-Edwards, 1869)	**KT365594**	NA	**KT425049**	NA		UF13466
*Charybdis anisodon (De Haan, 1850)*	**KT365536**	NA	NA	NA	A	UF11429
*Charybdis annulata* (Fabricius, 1798)	**KT365595**	**KT365708**	**KT425027**	**KT365634**	B	UF22076
*Charybdis bimaculata* (Miers, 1886)	**KT365596**	**KT365709**	**KT425036**	*JX398089		ZRC 2017.0508/ZMMUMa3396
*Charybdis callianassa* (Herbst, 1789)	**KT365537**	**KT365710**	**KT425035**	NA	A	ZRC1993.378-384
*Charybdis feriata* (Linnaeus, 1758)	**KT365538**	**KT365712**	**KT425051**	**KT365636**	A, B	UF3739
*Charybdis granulata* (De Haan, 1833)	NA	JX398102	JX398118	JX398090	C	NA
*Charybdis hellerii* (A. Milne-Edwards, 1867)	**KT365540**	**KT365715**	**KT424999**	**KT365638**	A, B	UF11430
*Charybdis hongkongensis* Shen, 1934	NA	JX398100	JX398112	JX398088	C	ZMMUMa3363
*Charybdis japonica* (A. Milne-Edwards, 1861)	FJ460517	*KT365716	*KT425042	NA	A	NA/*ZRC2008.0567
*Charybdis longicollis* Leene, 1938	**KT365541**	**KT365717**	**KT425054**	NA	A	UF3179
*Charybdis lucifera* (Fabricius, 1798)	**KT365542**	*KT365718	*KT425034	*KT365639	A, B	UF7667/*UF7684
*Charybdis natator* (Herbst, 1794)	**KT365543**	**KT365719**	*KT424998	NA	A	UF3707/*UF21403
*Charybdis orientalis* Dana, 1852	**KT588234**	**KT588225**	**KT781074**	NA		USNM112062
*Charybdis rathbuni* Leene, 1938	**KT365599**	**KT365722**	**KT425056**	NA		UF25655
*Charybdis sagamiensis* Parisi, 1916	**KT365598**	**KT365721**	NA	**KT365641**	B	UF29479
*Charybdis variegata* (Fabricius, 1798)	**KT365600**	**KT365723**	**KT425043**	NA		ZRC2012.1115
*Cronius edwardsii* (Lockington, 1877)	FJ152147	*KT588227	NA	NA	A	ULLZ8673/*USNM112311
*Cronius ruber* (Lamarck, 1818)	**KT365546**	*KT365725	**KT425008**	**KT365642**	A, B	UF26364/*UF25995
*Gonioinfradens paucidentatus* (A. Milne-Edwards, 1861)	**KT365547**	**KT365726**	*KT588216	NA	A	UF5109/*UF30184
*Goniosupradens acutifrons* (De Man, 1879)	**KT365535**	*KT365707	*KT425033	*KT365633	A, B	UF7114/*UF17047
*Goniosupradens erythrodactylus* (Lamarck, 1818)	**KT365597**	**KT365711**	NA	**KT365635**	B	UF1398
*Goniosupradens hawaiensis* (Edmondson, 1954), comb. nov.	**KT365539**	**KT365714**	**KT425023**	**KT365637**	A, B	UF25871
*Goniosupradens obtusifrons* (Leene, 1937)	**KT365544**	**KT365720**	**KT425007**	**KT365640**	A, B	UF16599
*Lissocarcinus arkati* Kemp, 1923	**KT365549**	**KT365729**	**KT425045**	**KT365643**	A, B	UF36296
*Lissocarcinus holothuricola* (Streets, 1877)	**KT365551**	**KT365731**	**KT425041**	**KT365645**	A, B	UF30203
*Lissocarcinus laevis* Miers, 1886	**KT365550**	**KT365730**	*KT425020	*KT365644	A, B	UF204/*UF39136
*Lissocarcinus orbicularis* Dana, 1852	**KT365552**	**KT365732**	*KT425032	NA	A	UF15741/*UF15429
*Lissocarcinus polybiodes* Adams & White, 1849	**KT365602**	**KT365733**	**KT424994**	**KT365646**	B	UF35245
*Thalamita admete* (Herbst, 1803)	**KT365562**	*KT365749	*KT425014	*KT365658	A, B	UF7688/*UF16971
*Thalamita* aff. *admete*	**KT365561**	**KT365748**	**KT424995**	**KT365657**	A, B	UF17745
*Thalamita auauensis* Rathbun, 1906	**KT365563**	**KT365750**	**KT425022**	NA	A	UF12320
*Thalamita bevisi* (Stebbing, 1921)	**KT365564**	**KT365751**	**KT425048**	**KT365659**	A, B	UF197
*Thalamita bouvieri* Nobili, 1906	**KT365565**	**KT365752**	*KT425016	**KT365660**	A, B	UF24801/*UF17562
*Thalamita chaptalii* (Audouin, 1826)	**KT365568**	**KT365758**	*KT425047	*KT365663	A, B	UF13103/*UF206
*Thalamita* cf. *gatavakensis* sp. A	**KT365576**	**KT365767**	**KT424997**	**KT365670**	A, B	UF16649
*Thalamita* cf*. gatavakensis* sp. B	**KT365575**	*KT365766	**KT424992**	**KT365669**	A, B	UF17469/*UF17486
*Thalamita gloriensis* Crosnier, 1962	**KT365582**	**KT365779**	**KT425038**	**KT365678**	A, B	UF25902
*Thalamita granosimana* Borradaile, 1902	**KT365577**	**KT365769**	**KT425005**	**KT365671**	A, B	UF24790
*Thalamita integra* Dana, 1852	**KT365578**	*KT365770	*KT425028	*KT365672	A, B	UF587/*UF22085
*Thalamita kagosimensis* Sakai, 1939	**KT365612**	**KT365771**	**KT425011**	**KT365673**	B	ZRC 2017.0514
*Thalamita* aff. *kukenthali*	**KT365608**	**KT365753**	**KT425052**	NA		UF33634
*Thalamita malaccensis* Gordon, 1938	**KT365614**	**KT365774**	**KT425010**	NA		ZRC 2017.0512
*Thalamita mitsiensis* Crosnier, 1962	**KT365580**	**KT365775**	*KT425053	**KT365675**	A, B	UF21937/*UF190
*Thalamita oculea* Alcock, 1899	**KT365616**	**KT365777**	**KT425044**	NA		ZRC 2017.0513
*Thalamita parvidens* (Rathbun, 1907)	**KT365567**	**KT365757**	**KT425037**	**KT365662**	A, B	UF17595
*Thalamita philippinensis* Stephenson & Rees, 1967	**KT365579**	**KT365772**	**KT425006**	**KT365674**	A, B	UF24920
*Thalamita picta* Stimpson, 1858	**KT365581**	**KT365778**	**KT425013**	**KT365677**	A, B	UF24881
*Thalamita pseudoculea* Crosnier, 1984	**KT365610**	**KT365754**	**KT425050**	NA		UF13877
*Thalamita pseudopoissoni* Stephenson & Rees, 1967	**KT365609**	**KT365755**	**KT425055**	NA		UF5051
*Thalamita quadrilobata* Miers, 1884	**KT365585**	**KT365782**	*KT425015	*KT365680	A, B	UF14254/*UF14608
*Thalamita savignyi* A. Milne-Edwards, 1861	**KT365618**	**KT365784**	**KT425061**	**KT365682**	B	UF7689
*Thalamita seurati* Nobili, 1906	**KT365587**	**KT365785**	**KT425004**	**KT365683**	A, B	UF12832
*Thalamita sima* H. Milne Edwards, 1834	**KT365619**	**KT365786**	*KT588217	**JX398086		UF35869/*UF36191/**ZMMUMa3373
*Thalamita aff. spinifera*	**KT365621**	**KT365788**	**KT425001**	NA		UF33379
*Thalamita stephensoni* Crosnier, 1962	**KT365623**	**KT365790**	**KT425059**	NA		UF17070
*Thalamitoides quadridens* A. Milne-Edwards, 1869	**KT365588**	*KT365792	**KT425017**	NA	A	UF18495/*UF15637
*Thalamitoides spinigera* Nobili, 1905	**KT365625**	**KT365793**	NA	**KT365687**	B	UF32881
*Thalamitoides tridens* A. Milne-Edwards, 1869	**KT365626**	**KT365794**	NA	**KT365688**	B	UF18231
*Thalamonyx gracilipes* A. Milne-Edwards, 1873	**KT365611**	**KT365768**	**KT425000**	NA		USNM274300
*Thranita coeruleipes* (Hombron & Jacquinot, 1846), comb. nov.	**KT365569**	**KT365759**	**KT425057**	**KT365664**	A, B	UF3232
*Thranita crenata* (Rüppell, 1830), comb. nov.	**KT365572**	**KT365763**	*KT424991	**JX398085/*KT365667	A, B	UF8950/*UF17752/**ZMMUMa3343
*Thranita danae* (Stimpson, 1858), comb. nov.	**KT365573**	*KT365764	*KT425031	**KT365668**	A, B	UF22114/*UF25992
*Thranita foresti* (Crosnier, 1962), comb. nov.	**KT365574**	**KT365765**	**KT425040**	NA	A	UF2222
*Thranita* cf. *prymna* (Herbst, 1803), comb. nov.	**KT365583**	**KT365780**	**KT425025**	*JX398084	A	UF14613/*ZMMUMa3346
*Thranita pseudopelsarti* (Crosnier, 2002), comb. nov.	**KT365584**	**KT365781**	**KT425039**	**KT365679**	A, B	UF16218
*Thranita rubridens* (Apel & Spiridonov, 1998), comb. nov.	**KT365586**	**KT365783**	**KT425060**	**KT365681**	A, B	UF7700
*Thranita* aff*. rubridens*	**KT365566**	**KT365756**	**KT425021**	**KT365661**	A, B	UF25803
*Thranita spinicarpa* (Wee & Ng, 1995), comb. nov.	**KT365620**	**KT365787**	**KT425012**	**KT365684**	B	UF36225
*Thranita spinimana* (Dana, 1852), comb. nov.	**KT365622**	**KT365789**	NA	**KT365685**	B	UF36209
*Trierarchus* cf. *cooperi* sp. A, comb. nov.	**KT365570**	**KT365760**	**KT424996**	**KT365665**	A, B	UF16152
*Trierarchus* cf. *cooperi* sp. B. comb. nov.	**KT365571**	**KT365761**	**KT425029**	**KT365666**	A, B	UF16949
*Trierarchus rotundifrons* (A. Milne-Edwards, 1869), comb. nov.	**KT365530**	**KT365698**	*KT424989	*KT365630	A, B	UF4079/*UF4057
*Trierarchus squamosus* (Stephenson & Hudson, 1957), comb. nov.	**KU737571**	NA	NA	NA		USNM102963
*Trierarchus woodmasoni* (Alcock, 1899), comb. nov.	**KT365624**	**KT365791**	**KT425026**	**KT365686**	B	UF4114
*Zygita longifrons* (A. Milne-Edwards, 1869), comb. nov.	**KT365613**	**KT365773**	**KT425002**	NA		UF7343
*Zygita murinae* (Zarenkov, 1971), comb. nov.	**KT365615**	**KT365776**	**KT425018**	**KT365676**	B	UF36525

**Notes:**

Bolded Genbank numbers represent data generated for this study, for voucher locality data and source references of all other sequences see Table S1.

* and ** Associated attributes for second and third specimens, respectively, in multi-specimen operational taxonomic units (OTUs).

A, 16S rRNA data include tRNA-Leu and partial NADH1 sequences.

B, 28S rRNA sequences > 500 bps and were included in analyses of 28S only data.

C, included only in single marker and 174 OTU concatenated analyses.

Voucher prefixes refer to the following institutions: BYU, Monte L. Bean Life Science Museum, Brigham Young University, Provo; CCDB, Crustacean Collection of the Department of Biology, University of São Paulo, São Paulo; CSIRO, CSIRO Marine Research collections, Hobart; MB, Museu Nacional de Historia Natural, Universidade de Lisboa, Lisbon; MNHN, Muséum National d’Histoire Naturelle, Paris; MZUCR, Zoology Museum, Universidad de Costa Rica, San José; MZUF, La Specola, Museo Zoologico Universita di Firenze, Florence; NMMBCD, National Museum of Marine Biology and Aquarium, Taiwan; NTOU, National Taiwan Ocean University, Keelung; SMF, Senckenberg Research Institute and Natural History Museum in Frankfurt; UF, Florida Museum of Natural History, University of Florida, Gainesville; ULLZ, Zoological Collection, University of Louisiana at Lafayette, Lafayette; USNM, Smithsonian National Museum of Natural History, Washington; ZMMU, Zoological Museum of the Moscow University, Moscow; ZRC, the Zoological Reference Collection of the Lee Kong Chian Natural History Museum, Singapore.

### Morphological terminology

**Figure 6:**
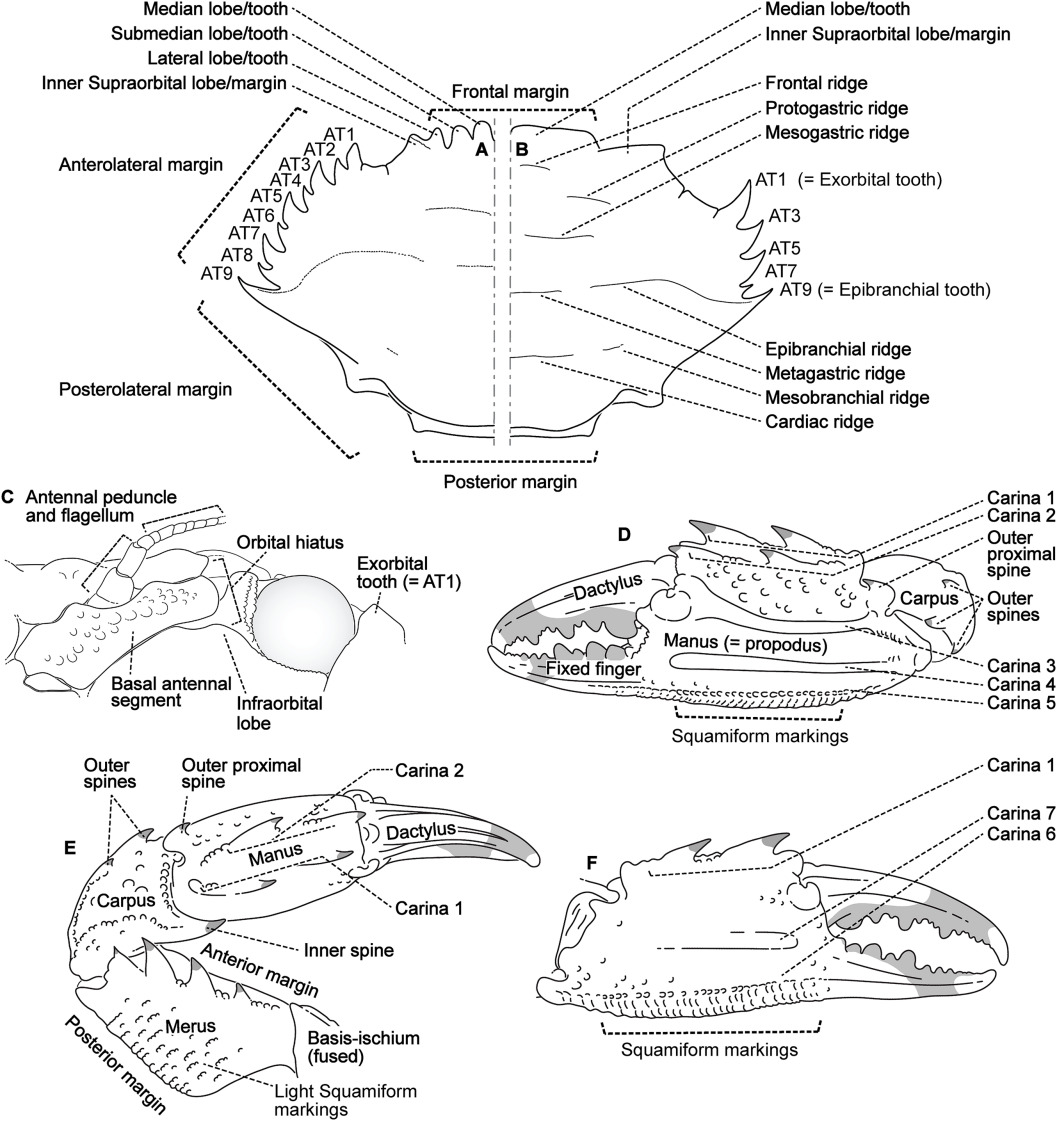
Morphological terminology for the carapace, antenna, and cheliped. Carapace dorsal surface: (A) *Cronius edwardsii* (USNM 1254607); (B) *Thalamita gatavakensis* (UF 24660). (C) Stylized ventral surface of antenna and orbit. (D–F) Stylized thalamitine left cheliped: (D), outer surface; (E) dorsal surface; (F) inner surface. AT, positionally homologous portunid anterolateral tooth number.

**Figure 7:**
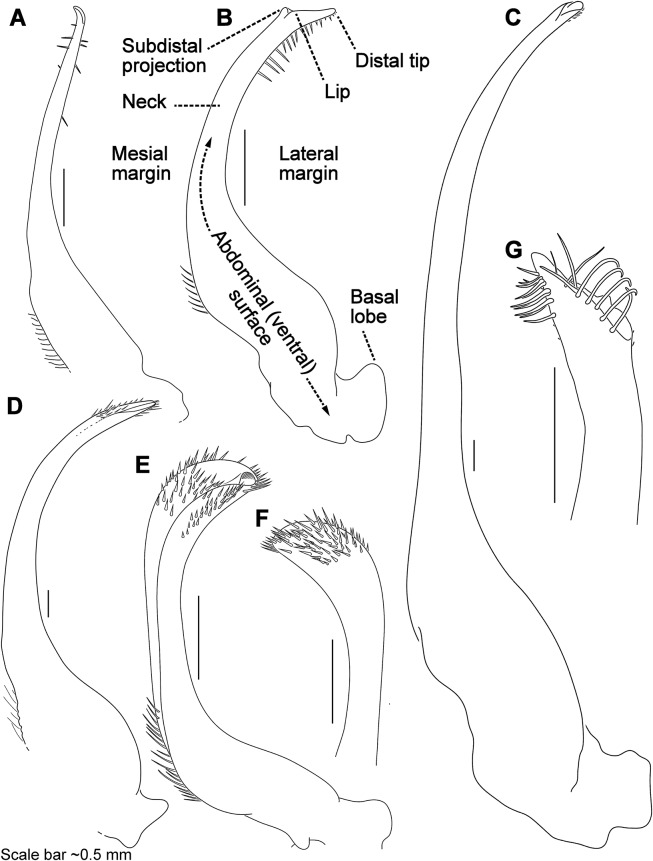
Morphology and terminology for stylized left male first gonopod (G1) from representative taxa. (A) *Thalamita granosimana* (*Thalamita* sensu stricto clade 1; composite redrawn from Stephenson & Rees, 1967a, Fig. 27A and 27B); (B) *Thalamita spinifera* (*Thalamita* sensu lato “clade” II; redrawn from Crosnier, 1962, Fig. 214); (C) *Thranita crenata* (“*Thalamita”* sensu lato clade III; composite redrawn from Crosnier, 1962, Figs. 232 and 233); (D) *Zygita murinae* (composite redrawn from Spiridonov & Neumann, 2008, Figs. 6 and 7); (E, F) *Trierarchus woodmasoni* (redrawn from Crosnier, 1975a, Figs. 8J and 8I, respectively); (G) *Thalamonyx gracilipes* (redrawn from Stephenson & Rees, 1967b, Fig. 2H). (A–E) depict the abdominal (ventral) G1 surface, (F, G) depict distal portion of the sternal (dorsal) G1 surface.

Descriptive work on portunoid crabs has not always used consistent morphological terminology. Morphological terms used here are illustrated in Figs. 4, 6 and 7, and mostly conform to those used by Apel & Spiridonov (1998), Crosnier (1962), Stephenson & Hudson (1957), and Wee & Ng (1995). As in these works, here the demarcation of teeth (or lobes) along the frontal margin of the carapace does not include the inner supraorbital margins, but discussion (or counts) of the teeth along the anterolateral margins does include the exorbital tooth (as tooth number one; Figs. 6A and 6B). Standard pereiopod abbreviations are also followed: P1, cheliped; P2–P4, ambulatory legs; P5, natatory (swimming) leg (Fig. 4). Likewise, G1 and G2 denote male first and second gonopods, respectively (Fig. 7).

**Figure 8:**
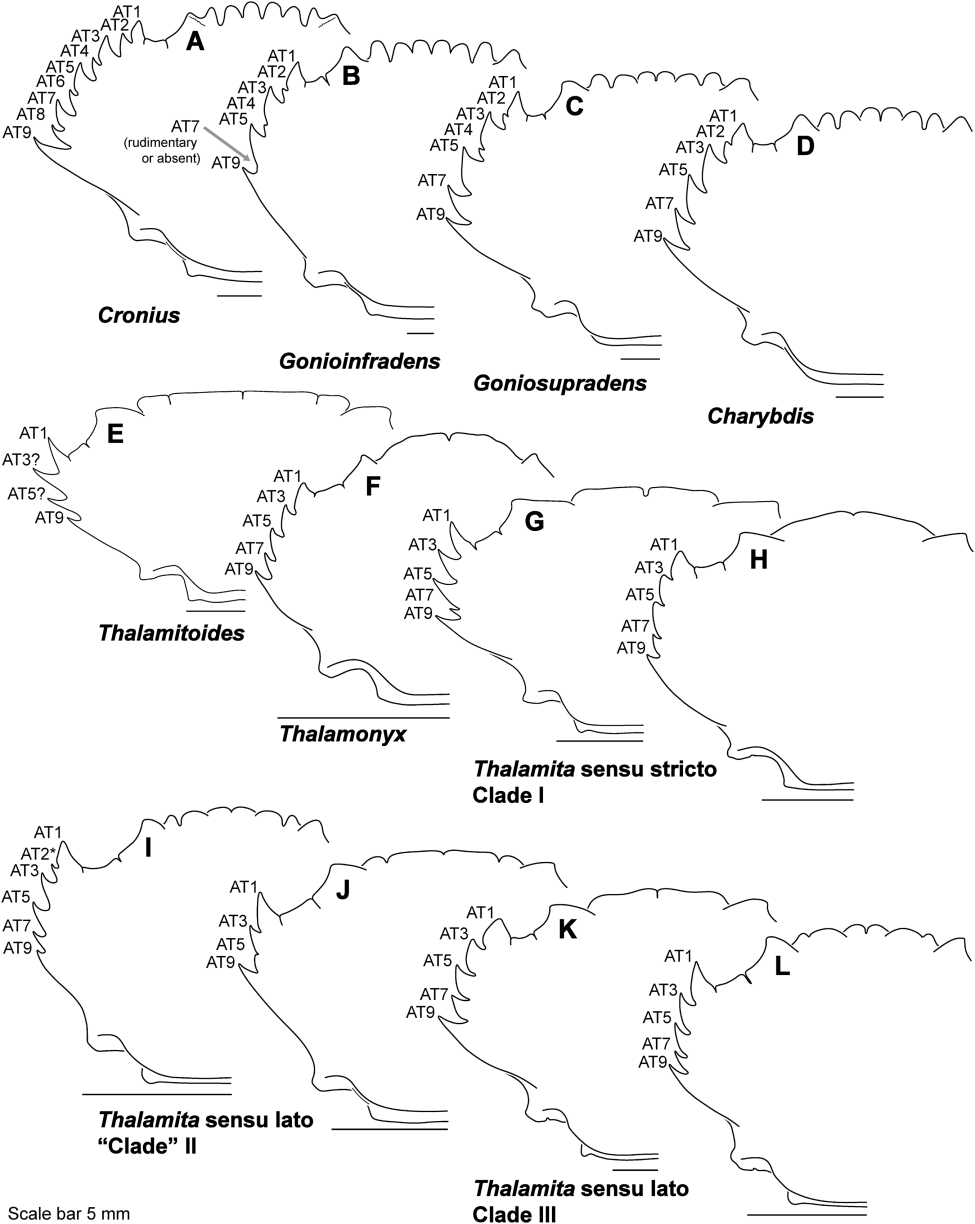
Representative partial carapace outlines of Thalamitinae genera, Part 1. (A) *Cronius edwardsii* (USNM 1254607); (B) *Gonioinfradens paucidentatus* (UF 1411-A); (C) *Goniosupradens obtusifrons* (UF 16599); (D) *Charybdis orientalis* (USNM 112062); (E) *Thalamitoides quadridens* (UF 1962); (F) *Thalamonyx gracilipes* (USNM 127103-A); (G) *Thalamita admete* (UF 26950-A); (H) *Thalamita parvidens* (USNM 32855-A; Holotype); (I) *Thalamita spinifera* (UF 33379); (J) *Thalamita bouvieri* (UF 41652); (K) *Thalamita sima* (USNM 1254584-A); (L) *Thalamita malaccensis* (USNM 274290-A). AT, positionally homologous portunid anterolateral tooth number (see Figs. 6A and 6B and text). Asterisks indicate a homoplastic anterolateral tooth that arose through parallelism or reversal (see text).

**Figure 9:**
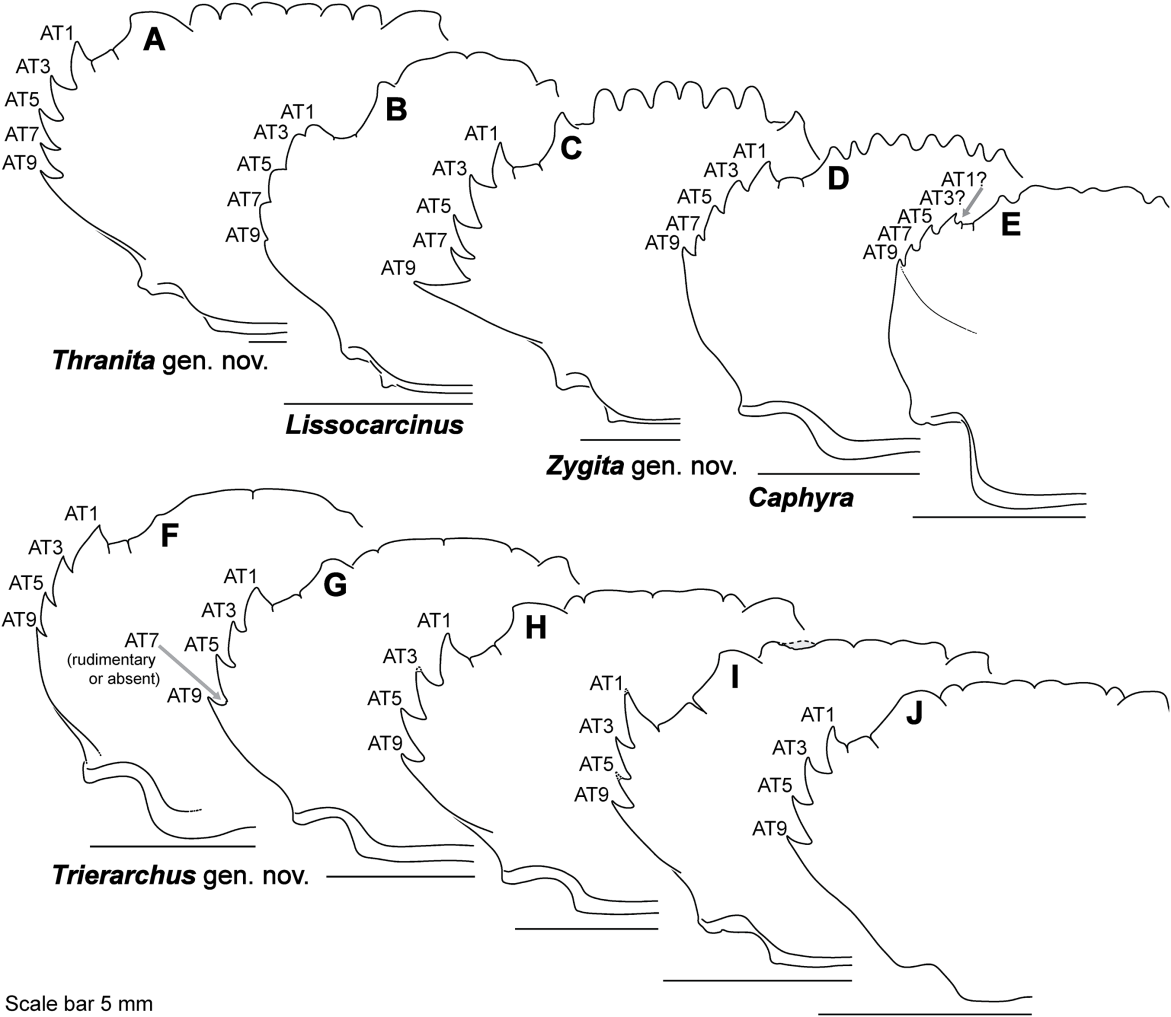
Representative partial carapace outlines of Thalamitinae genera, Part 2. (A) *Thranita crenata,* comb. nov. (UF 39965); (B) *Lissocarcinus laevis* (UF 41571); (C) *Zygita longifrons,* comb. nov. (UF 199); (D) *Caphyra loevis* (UF 38881); (E) *Caphyra* cf. *fulva* (UF 38855; epibranchial ridge depicted); (F) *Trierarchus rotundifrons*, comb. nov. (UF 40143-A); (G) *Trierarchus woodmasoni*, comb. nov. (UF 40079); (H) *Trierarchus cooperi* sp. B, comb. nov. (USNM 41125-A); (I) *Trierarchus squam*osus, comb. nov. (USNM 102963); (J) *Trierarchus acanthophallus,* comb. nov. (stylized outline redrawn from Chen & Yang, 2008). AT, positionally homologous portunid anterolateral tooth number (see Figs. 6A and 6B and text).

Finally, here I propose new terminology in the form of two numbering schemes to respectively characterize carapace anterolateral teeth and cheliped carinae in Portunidae (Figs. 6A, 6B, 6D and 6F). In both cases, indicated structures clearly share positional homology across Portunidae (likely Portunoidea) and adoption of the proposed schemes should bring greater clarity to taxonomic descriptive work on portunids. For example, anterolateral teeth counts are often diagnostic for *Thalamita* where five teeth are standard, but the fourth is often absent and the first sometimes exhibits a subsidiary tooth. Confusion can arise when diagnoses of *Thalamita* discuss the form or presence of the “fourth tooth” in disparate species exhibiting a total of four, five or six anterolateral teeth (e.g., compare Figs. 8G–8J). Under the proposed scheme such confusion is avoided; the diagnostic “fourth” anterolateral tooth typically refers to portunid tooth AT7, and is better discussed as such in each of these cases. Likewise a simple count of spines on the upper surface of the cheliped can lead to confusing descriptions when standard spines are absent from different cheliped carinae for different taxa. Although a determination of positional homology for anterolateral teeth may be difficult for select taxa (e.g., Figs. 8E and 9E), “transitional” forms may significantly help. For example, while exhibiting nine anterolateral teeth is clearly plesiomorphic within Portunidae (Spiridonov, Neretina & Schepetov, 2014), in *Cronius* these teeth alternate in size such that each of its five large teeth are separated by a reduced (or subequal) tooth (Fig. 8A). This suggests that the five anterolateral teeth typical to *Thalamita* likely correspond (in order) to teeth numbers one, three, five, seven, and nine in portunine taxa (compare Figs. 6A and 6B). This is supported by additional intermediate forms present in other Thalamitinae taxa (Figs. 8B–8D). Last, it is worth noting that some positionally homologous anterolateral teeth are likely homoplastic, reappearing within derived clades through reversal or parallelism (e.g., AT2* in Fig. 8I).

### Nomenclatural acts

The electronic version of this article in portable document format will represent a published work according to the International Commission on Zoological Nomenclature (ICZN), and hence the new names contained in the electronic version are effectively published under that Code from the electronic edition alone. This published work and the nomenclatural acts it contains have been registered in ZooBank, the online registration system for the ICZN. The ZooBank LSIDs (Life Science Identifiers) can be resolved and the associated information viewed through any standard web browser by appending the LSID to the prefix http://zoobank.org/. The LSID for this publication is: urn:lsid:zoobank.org:pub:90E97894-9BBE-452C-A6A8-AFF7C1B78874. The online version of this work is archived and available from the following digital repositories: PeerJ, PubMed Central and CLOCKSS.

### DNA extractions, amplification and sequencing

Molecular work was conducted at the Florida Museum of Natural History and the Smithsonian Institution’s Laboratories of Analytical Biology. DNA was extracted using a standard phenol–chloroform protocol by hand or on an Autogen Prep 956 Extractor (AutoGen Inc., Holliston, MA, USA). A total of 345 sequences from four molecular markers (16S rRNA, CO1, 28S rRNA, and H3) were generated from 114 portunoid species, 76 of which have never before been sequenced. Amplifications were carried out following protocols outlined in Evans & Paulay (2012), Lasley, Klaus & Ng (2015), and Leray & Knowlton (2015). Typically this included the use of a “step-down” PCR profile (Evans & Paulay, 2012). This approach involves using a higher annealing temperature for the first five PCR cycles followed by 30 cycles at a lower annealing temperature. Table 2 lists primer pairs, annealing temperatures and resulting fragment sizes for each marker. Amplification of 16S rRNA resulted in at least 500 bps of sequence, but one primer set yielded a 1.2 kb fragment that includes tRNA-Leu and partial NADH1. Both 16S fragments were combined into a single data set that, unless otherwise stated, is referred to here as 16S data (fragment distinctions indicated in Table 1 notes). Clean up, cycle sequencing and purification were carried out on all successful PCR products using Exosap-It (Affymetrix Inc., Santa Clara, CA, USA), ABI BigDye terminator V3.1 reactions and a Sephadex G-50 protocol. Resulting products were bidirectionally sequenced on an ABI 3130xl genetic analyzer (Applied Biosystems, Foster City, CA, USA). Consensus sequences were generated using Geneious v. 7.1.8 (Kearse et al., 2012) and submitted to GenBank. GenBank accession numbers are listed in Table 1.

**Table 2:** Primer pairs, annealing temperatures and resulting fragment sizes for PCR reactions.

Primer pairs—forward/reverse	5′–3′ Forward primer sequence	5′–3′ Reverse primer sequence	*T*_a_ (°C)	Approximate amplicon size (bps)	Reference
**CO1**					
dgLCO1490/dgHCO2198	GGTCAACAAATCATAAAGAYATYGG	TAAACTTCAGGGTGACCAAARAAYCA	50 and 45	650	Meyer (2003)
jgLCO1490/jgHCO2198	TITCIACIAAYCAYAARGAYATTGG	TAIACYTCIGGRTGICCRAARAAYCA	50 and 45	650	Geller et al. (2013)
**16S rRNA + tRNA-Leu + NADH1**					
NDH5/16L2	GCYAAYCTWACTTCATAWGAAAT	TGCCTGTTTATCAAAAACAT	48 and 44	1,200	Schubart, Cuesta & Felder (2002)
**16S rRNA**					
16H11/16L2	AGATAGAAACCRACCTGG	See above	48 and 44	580	Schubart (2009)
crust16sF1/crust16sR2	CCGGTYTGAACTCAAATCATGTAAA	TTGCCTGTTTATCAAAAACATGTYTRTT	50 and 45	515	Lai et al. (2009)
**28S (D1–D2 region)**					
LSUfw1brach/LSUrev1brach	AGCGGAGGAAAAGAAACYA	TACTAGATGGTTCGATTAGTC	50 and 45	1,300	This study*
LSUfw2brach/LSUrev2brach	ACAAGTACCGTGAGGGAAAGTTG	ACAATCGATTTGCACGTCAG	55 and 50	890	This study*
F635/LSUrev2brach	CCGTCTTGAAACACGGACC	See above	55 and 50	600	Medina et al. (2001)
**H3**					
H3af/H3ar	TGGCTCGTACCAAGCAGACVGC	TATCCTTRGGCATRATRGTGAC	50 and 47	327	Colgan et al. (1998)

**Notes:**

* Modified from Sonnenberg, Nolte & Tautz (2007).

*T*_a_, annealing temperatures used here in a “step-down” PCR approach (see text).

### Taxon sampling and composition of molecular data sets

A molecular data set comprised of 174 operational taxonomic units (OTUs) was constructed for this study. This data set combined 344 newly generated sequences with 176 previously published fragments of 16S rRNA, CO1, 28S rRNA, and H3 data. Published sequences were mostly drawn from recent phylogenetic studies on Portunoidea, including Mantelatto et al. (2009), Schubart & Reuschel (2009), and Spiridonov, Neretina & Schepetov (2014). With some exceptions, taxon sampling was designed to include portunoid lineages at or above the species-level, avoiding genetically and morphologically highly conserved species complexes, especially those previously investigated (e.g., *Callinectes* by Robles et al., 2007; *Portunus pelagicus* by Lai, Ng & Davie, 2010). The complete data set includes 168 ingroup portunoid taxa and six outgroup taxa. The relative position of Portunoidea within Brachyura remains poorly resolved (Tsang et al., 2014) so outgroup taxa were selected with reference to previous studies. Details of each OTU are listed in Table 1 and Table S1, including taxonomy, GenBank accession numbers, voucher information, and source publications. One hundred eight of these OTUs consist of sequences generated from a single vouchered specimen. For most of the remaining multi-specimen OTUs species-level matches were confirmed with additional newly generated or previously published CO1 or 16S rRNA data (including some unpublished DNA barcode data; analyses not shown). This approach permitted the inclusion of longer, more complete sequence data, but OTUs with missing data were unavoidable.

In an effort to mitigate the impact of missing data, two reduced concatenated data sets were also constructed from the original. The first included 163 taxa, representing all OTUs with at least 16S rRNA data. The second included 138 taxa, representing all OTUs with at least 16S rRNA and CO1 data. Additionally, each molecular marker was analyzed separately before concatenation, thus constituting four additional data sets. However, for the 28S rRNA only data set, just 66 of the total 85 sequences were included. This approach avoided all 28S sequences with less than 500 bps of data, most of which span the uninformative D1 region. Finally, preliminary analyses of 16S rRNA recovered the putative portunoid taxon *Brusinia profunda* falling far outside Portunoidea. Consequently, newly generated 16S rRNA data for this important taxon (voucher USNM 277519, GenBank KX425018, Fig. 1A) was not included in the above data sets. Instead, this 517 bps sequence was added to an additional “Brusinia-16S” data set that combined all 163 sequences from the 16S rRNA only portunoid data set and 145, mostly brachyuran, 16S rRNA sequences analyzed by Tsang et al. (2014). Taxon identity, GenBank numbers, and voucher IDs for all data used from Tsang et al. (2014) appear as taxon labels in the analyzed data set and resulting phylogeny. In summary, eight molecular data sets were constructed for phylogenetic analyses. Each data set is summarized in Table 3 including marker composition, alignment length and the number of parsimony informative sites.

**Table 3:** Composition of eight molecular data sets constructed for phylogenetic analyses.

Dataset name	Taxon sampling	Dataset composition	Alignment length (bps)	Parsimony informative sites (bps)
16S-only	163 taxa	16S rRNA	1,105	521
CO1-only	148 taxa	CO1	657	260
28S-only	66 taxa	28S rRNA D1–D2 region (>500 bps)	1,224	184
H3-only	123 taxa	H3	327	106
174 taxa concatenated	174 taxa	16S rRNA − 163 taxa/CO1 − 148 taxa/28S rRNA − 85 taxa/H3 – 123 taxa	3,313	1,080
163 taxa concatenated	163 taxa	16S rRNA − 163 taxa/CO1 − 138 taxa/28S rRNA − 74 taxa/H3 − 115 taxa	3,313	1,074
138 taxa concatenated	138 taxa	16S rRNA − 138 taxa/CO1 − 138 taxa/28S rRNA − 70 taxa/H3 − 103 taxa	3,313	1,039
Brusinia-16S	309 taxa	16S rRNA − 163 taxa (as above) + *Brusinia profunda* + 145 taxa (Tsang et al., 2014)	447	237

### Modified identifications of published sequences

Several published portunoid sequences appear to have been misidentified and were addressed as follows. The CO1 sequence data for *Charybdis natator* analyzed in Spiridonov, Neretina & Schepetov (2014) matched that of *Charybdis granulata* (GenBank KT365713; Voucher ZRC-2000.0771; Phuket, Thailand; specimen examined, identity confirmed) and not *Ch. natator* used in this study (Table 1). Consequently, CO1, H3 and 28S rRNA sequence data for *Ch. natator* from Spiridonov, Neretina & Schepetov (2014) were included in this study but identified as *Ch. granulata*. Likewise, phylogenetic analyses of H3 sequence data for *Thalamita sima* from Spiridonov, Neretina & Schepetov (2014; GenBank JX398122) strongly suggests that it represents contamination from a separate *Charybdis bimaculata* specimen. That is, this sequence matches that of *Ch. bimaculata* generated for this study and that from Spiridonov, Neretina & Schepetov (2014). This sequence was not included in this study. However, 28S data and CO1 data from this specimen (GenBank JX398086 and JX398105, respectively) are not similarly suspect. A comparison of CO1 data with additional newly generated sequences for *Th. sima* (GenBank KT588224 and KT365786) confirm that Spiridonov, Neretina & Schepetov (2014) collected and sequenced a correctly identified *Th. sima* specimen.

### Sequence alignment and phylogenetic analyses

Sequence alignments were constructed using MAFFT v 7.123b (Katoh & Standley, 2013) under the E-INS-i setting. Unreliably aligned columns for 16S and 28S rRNA data sets were identified and removed using Guidance2 (Sela et al., 2015), similarly employing MAFFT’s E-INS-i settings (–genafpair –maxiterate 1,000). Each Guidance2 run evaluated 400 alternative alignments generated from 100 alternative guide trees. Columns with a confidence score below 0.9 were trimmed from the final alignment. The *Brusinia*-16S data set was similarly aligned, but its total length was trimmed to just 447 bps, covering only those sites available in the 16S data of Tsang et al. (2014). Substitution models and partition schemes were evaluated for each data set using the BIC criterion and a greedy search algorithm in Partitionfinder v.1.1.1 (Lanfear et al., 2012). For each data set all models were evaluated as well as just the reduced set available in MrBayes (Ronquist et al., 2012). A single partition and a GTR+I+G model were chosen for the Brusinia-16S data set. The best scoring schemes for the remaining seven data sets are outlined in Table 4 and Table S2 and were used in subsequent partitioned phylogenetic analyses. Maximum likelihood (ML) phylogenetic analyses were carried out on all data sets using GARLI 2.0 (Zwickl, 2006). For each concatenated data set and the Brusinia-16S data set, ML analyses consisted of at least 100 independent searches and included both random and fast ML stepwise starting trees (attachmentspertaxon = 50, 100, or 2N+1). For single marker data sets at least 20 independent ML searches were performed with stepwise starting trees (attachmentspertaxon = 100). Nodal support for each of the best scoring ML topology was evaluated with 500 bootstrap replicates generated using the same tree search parameters. Bayesian analyses (BI) were performed on each concatenated data sets using MrBayes v3.2.5 (Ronquist et al., 2012). A standard MrBayes MCMC analysis (nruns = 2, nchains = 4) was run on each data set and lasted 25 million generations, sampling every 10,000 generations. An arbitrary burn-in value of 2.5 million generations was used for the 138 OTU and 163 OTU concatenated data sets. A higher burn-in value of seven million generations was needed for the 174 OTU concatenated data set. The standard deviation of split frequencies was confirmed to be less than 0.01 for each analysis. Convergence was further evaluated using Tracer v.1.6 (Rambaut et al., 2014) and included confirmation that each run attained ESS values greater than 200. All phylogenetic analyses were carried out on the CIPRES Science Gateway (Miller, Pfeiffer & Schwartz, 2010). FigTree v1.4.0 was used to visualize trees and generate resulting figures. Sequence alignments and phylogenetic results were deposited to TreeBASE (accessible at https://treebase.org/treebase-web/search/study/summary.html?id=21486).

**Table 4:** Best scoring partition schemes for three concatenated molecular data sets.

Marker	Marker subset	Alignment positions	174 taxa concatenated data				163 taxa concatenated data				138 taxa concatenated data			
			Model for ML runs	ML partition ID	Model for BI runs	BI partition ID	Model for ML runs	ML partition ID	Model for BI runs	BI partition ID	Model for ML runs	ML partition ID	Model for BI runs	BI partition ID
16S rRNA	16S rRNA	1–583	TVM+I+G	1	GTR+I+G	1	TVM+I+G	1	GTR+I+G	1	TVM+I+G	1	GTR+I+G	1
	tRNA-LEU	584–653	TVM+I+G	1	GTR+I+G	1	TVM+I+G	1	GTR+I+G	1	TVM+I+G	1	GTR+I+G	1
	ND1	654–1,105	GTR+I+G	2	GTR+I+G	2	GTR+I+G	2	GTR+I+G	2	TrN+I+G	2	GTR+I+G	2
CO1	Codon Pos. 1	1,106–1,762\3	SYM+I+G	3	SYM+I+G	3	SYM+I+G	3	SYM+I+G	3	SYM+I+G	3	SYM+I+G	3
	Codon Pos. 2	1,107–1,762\3	F81+I+G	4	F81+I+G	4	F81+I+G	4	F81+I+G	4	F81+I+G	4	F81+I+G	4
	Codon Pos. 3	1,108–1,762\3	GTR+I+G	5	GTR+I+G	5	GTR+I+G	5	GTR+I+G	5	GTR+I+G	5	GTR+I+G	5
28S rRNA,H3	D1 and D2 region	1,763–2,986	GTR+I+G	6	GTR+I+G	6	GTR+I+G	6	GTR+I+G	6	GTR+I+G	6	GTR+I+G	6
	Codon Pos. 1	2,988–3,313\3	SYM+I+G	3	SYM+I+G	3	TrNef+I	8	SYM+I+G	3	TrNef+I	8	SYM+I+G	3
	Codon Pos. 2	2,989–3,313\3	F81+I+G	4	F81+I+G	4	TrNef+I	8	JC+I	8	TrNef+I	8	JC+I	8
	Codon Pos. 3	2,987–3,313\3	GTR+I+G	7	GTR+I+G	7	GTR+G	7	GTR+G	7	GTR+G	7	GTR+G	7

## Results and Discussion

**Figure 10:**
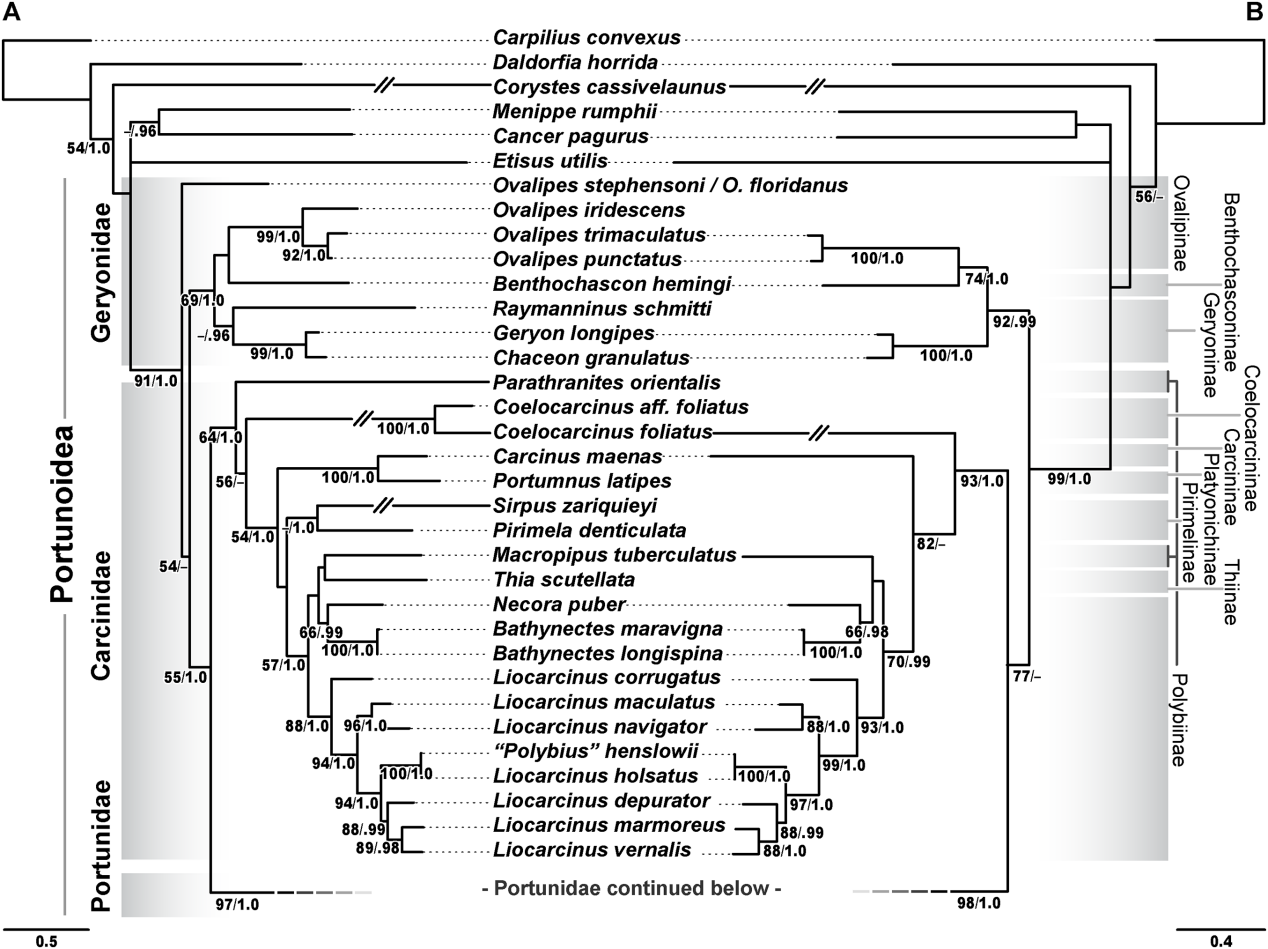
Maximum likelihood (ML) phylograms of Portunoidea based on analyses of 163 and 138 OTUs and a 3,313 bp alignment of 16S rRNA, CO1, 28S rRNA, and H3 sequence data, Part 1 (of 3). (A) ML phylogram based on analyses of 163 OTUs, each with at least 16S rRNA data; (B) ML phylogram based on analyses of 138 OTUs, each with at least 16S rRNA and CO1 data. Support values appear below relevant branches with ML bootstrap values ≥50% (based on 500 replicates) appearing first followed by BI posterior probabilities ≥0.95.

**Figure 11:**
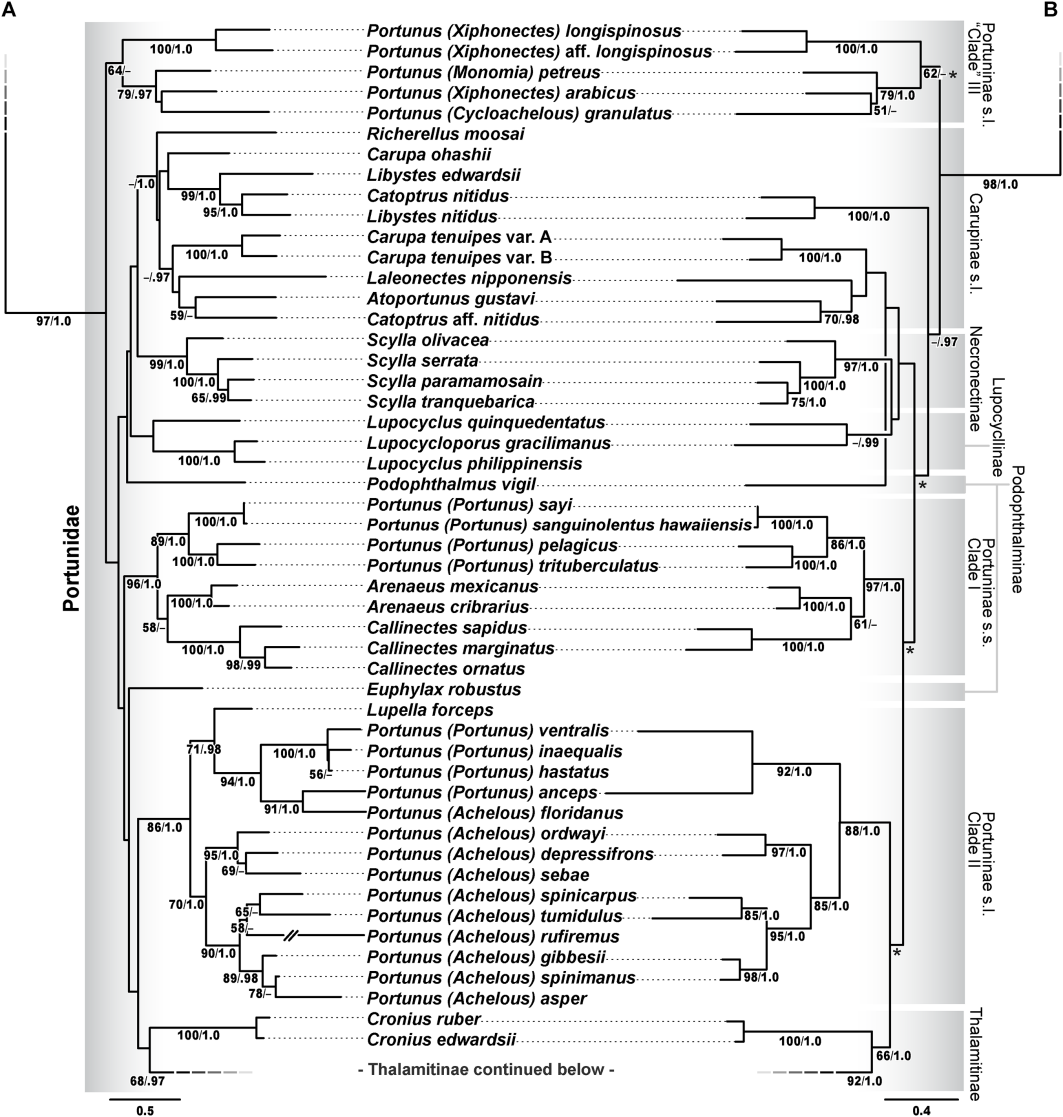
Maximum likelihood (ML) phylograms of Portunoidea based on analyses of 163 and 138 OTUs and a 3,313 bp alignment of 16S rRNA, CO1, 28S rRNA, and H3 sequence data, Part 2 (of 3). (A) ML phylogram based on analyses of 163 OTUs, each with at least 16S rRNA data; (B) ML phylogram based on analyses of 138 OTUs, each with at least 16S rRNA and CO1 data. Support values appear below relevant branches with ML bootstrap values ≥50% (based on 500 replicates) appearing first followed by BI posterior probabilities ≥0.95. Asterisk* denotes nodes that topologically conflict with corresponding BI topology (see text and Fig. 13B).

**Figure 12:**
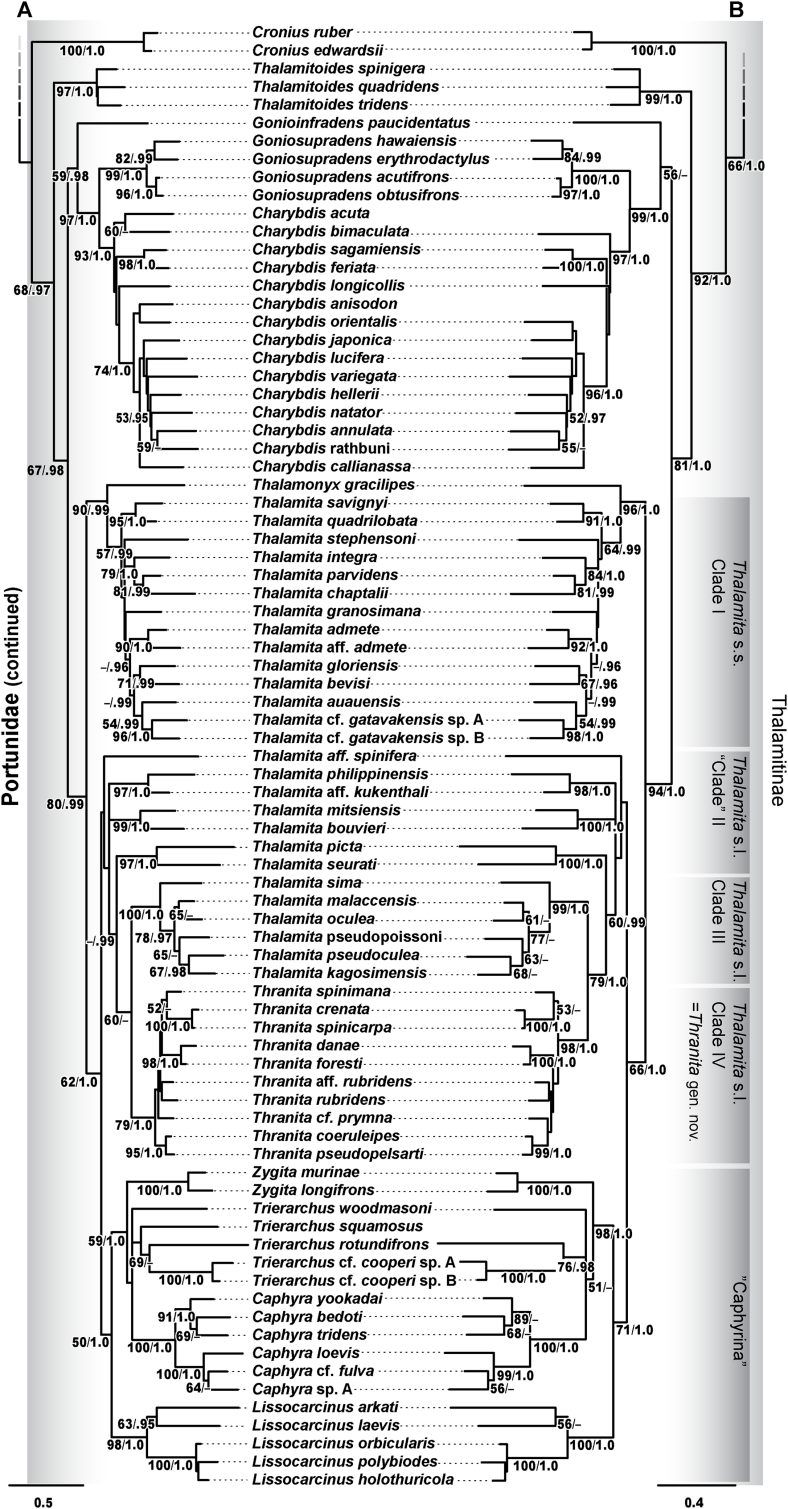
Maximum likelihood (ML) phylograms of Portunoidea based on analyses of 163 and 138 OTUs and a 3,313 bp alignment of 16S rRNA, CO1, 28S rRNA, and H3 sequence data, Part 3 (of 3). (A) ML phylogram based on analyses of 163 OTUs, each with at least 16S rRNA data; (B) ML phylogram based on analyses of 138 OTUs, each with at least 16S rRNA and CO1 data. Support values appear below relevant branches with ML bootstrap values ≥50% (based on 500 replicates) appearing first followed by BI posterior probabilities ≥0.95.

**Figure 13:**
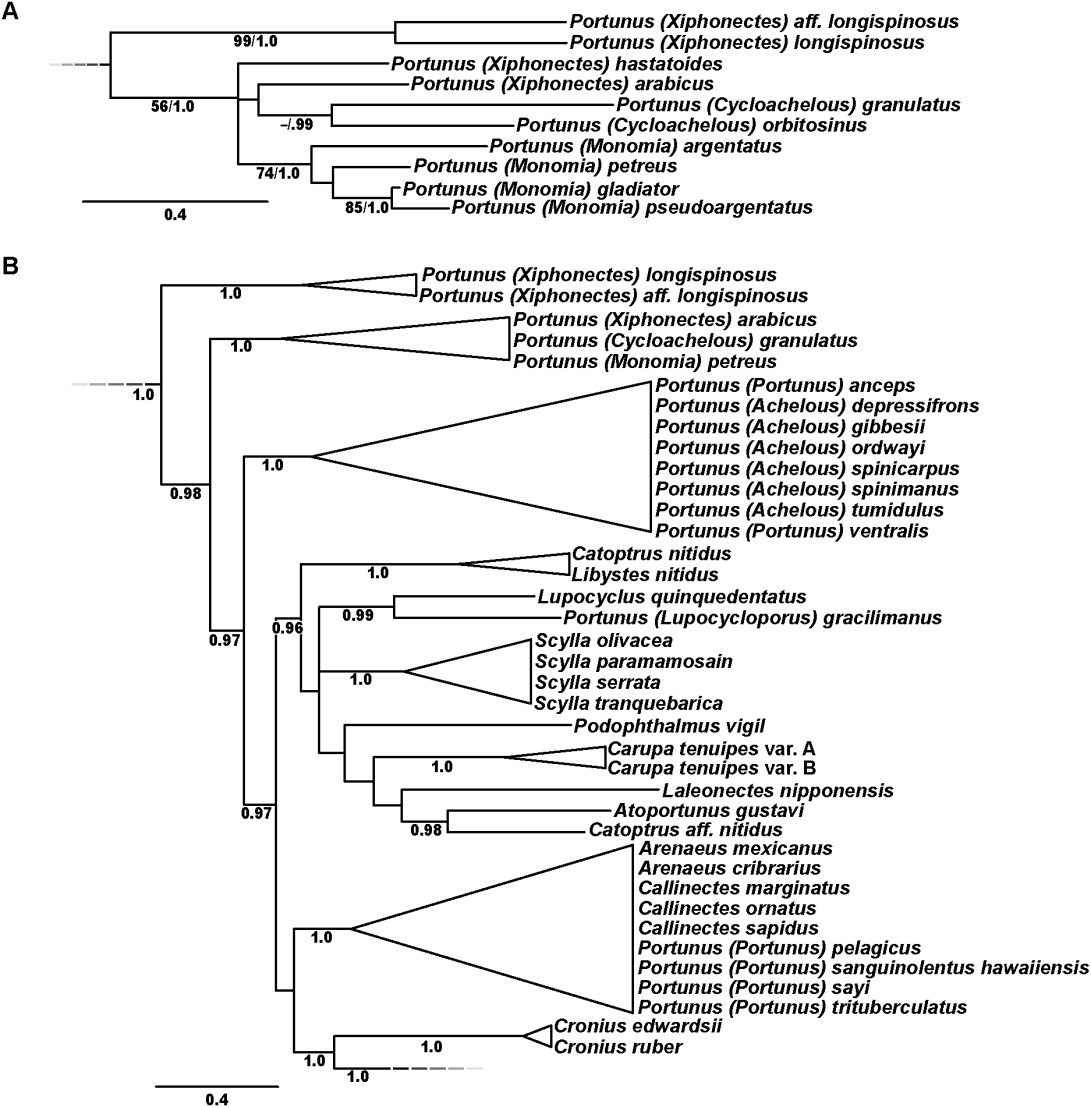
Subsections of ML and BI topologies for Portunoidea based on analyses of 174 and 138 OTUs and a 3,313 bp alignment of 16S rRNA, CO1, 28S rRNA, and H3 sequence data. (A) A subsection of the 174 OTU ML phylogram representing the *Portunus* subgenera *Cycloachelous, Monomia, and Xiphonectes*. Support values appear below relevant branches with ML bootstrap values ≥50% (based on 500 replicates) appearing first followed by BI posterior probabilities ≥0.95. (B) Relevant subsection of the 138 OTU BI majority consensus tree exhibiting topological conflict with the ML phylogram generated from the same concatenated data set (see text and Fig. 11). BI posterior probabilities (≥0.95) appear below each relevant node.

Phylogenetic analyses of up to four molecular markers (16S rRNA, CO1, 28S rRNA, and H3) were carried out on 168 portunoid OTUs, 76 for the first time. Resulting topologies and support values are summarized in Figs. 10–13 and Figs. S1–S6. With few exceptions phylogenetic analyses of the three concatenated data sets recovered consistent topologies that displayed significant support for most of the same clades (Figs. 10–12; Fig. S1). However, analyses of the 174 OTU data set, which had the greatest proportion of missing data, often recovered lower support for each clade (Fig. S1). Clades typically exhibited the greatest support in analyses of the 138 OTU data set, which contained the least amount of missing data (Figs. 10B, 11B and 12B). Nevertheless some topological incongruence was recovered between ML and BI analyses of this 138 OTU concatenated data set (compare nodal asterisks, Figs. 11B and 13B). This conflict was associated with deeper nodes in Portunidae and involved the relative placement of a well-supported “*Achelous*” sensu lato clade (discussed below). This conflict may be an artifact of the low taxon sampling available for non-thalamitine portunids, a general shortcoming in all analyses. Single marker ML analyses generally recovered poorly resolved topologies, but displayed no significant well-supported conflict with concatenated results (Figs. S2–S5). The following sections present a clade-by-clade discussion of the results for the ML and BI analyses of the 163 and 138 OTU concatenated data sets. The ML topologies for these two data sets are presented together in Figs. 10–12. In text, bootstrap support values (bs) and Bayesian posterior probabilities (pp) are reported together with those for the 163 OTU topology appearing first, followed by those for the 138 OTU topology (e.g., Fig. 10; bs 70%, 100%, pp 0.95, 1.0). Results of the other analyses, including those for the 16S-*Brusinia* data set, are discussed where relevant.

### Superfamily Portunoidea Rafinesque, 1815

Analyses recovered a strongly supported monophyletic Portunoidea (Fig. 10; bs 91%, 99%, pp 1.0, 1.0) comprised of three major, moderately well supported lineages (but see discussion regarding *Ovalipes*). These three lineages include taxa from seven of the eight currently valid portunoid families, and their relative composition is consistent with, but display greater resolution than that recovered in Schubart & Reuschel (2009) and Spiridonov, Neretina & Schepetov (2014). Summarizing these previous works, Davie, Guinot & Ng (2015a) suggested that the composition and status of each portunoid family may need to be reappraised, but only after all genera have been considered. However, given a shared morphology (discussed in detail by Davie, Guinot & Ng (2015b), Guinot, Tavares & Castro (2013), Karasawa, Schweitzer & Feldmann (2008) and Spiridonov, Neretina & Schepetov (2014)), and in light of the results presented below, the current number of valid portunoid families appears overstated. Here I propose a more conservative classification scheme for Portunoidea comprised of three instead of eight extant families: Geryonidae, Carcinidae, and Portunidae (Figs. 5 and 10). Included in this proposal, results discussed below also suggest that Brusiniidae Števčić (1991), is still a valid brachyuran family, but that it may not be a member of Portunoidea.

Family Geryonidae Colosi, 1923

The portunoid family Geryonidae *sensu* Schubart & Reuschel (2009) was recovered here as a well-supported clade comprised of *Benthochascon*, *Chaceon*, *Geryon*, *Ovalipes*, and *Raymanninus* (Fig. 10; bs 69%, 92%, pp 1.0, 0.99). These results challenge recent actions taken by Spiridonov, Neretina & Schepetov (2014) in which *Ovalipes* was removed from Geryonidae and the new family Ovalipidae established. Here I propose a more conservative classification in which *Ovalipes* is retained within Geryonidae in the subfamily Ovalipinae, status nov. (Fig. 5). However, further study is needed as both the 174 and 163 OTU concatenated analyses recovered a poorly supported placement of the hybrid OTU *Ovalipes stephensoni + Ovalipes floridanus* as sister to all other portunoids, thus rendering *Ovalipes* polyphyletic and Geryonidae paraphyletic (Fig. 10A; Fig. S1). This placement should be approached with caution and may very well be artifactual. That is, this placement is clearly unstable and was based on limited 16S and 28S rRNA data (461 bps and 618 bps, respectively). Furthermore, this OTU’s relative placement is also poorly resolved in both single gene analyses (Figs. S2 and S4), but was recovered with *Raymanninus* (with nominal support) as sister to all other *Ovalipes* in the *Brusinia-*16S ML analyses (Fig. S6). Nevertheless, the relative placement of this OTU is taxonomically important. Morphologically *O. stephensoni* and *O. floridanus* are sister species most closely related with the unsampled generic type *O. ocellatus* (Herbst, 1799) (see cladistic analyses of Parker, Mckenzie & Ahyong (1998)). If additional work finds further support for the polyphyly of *Ovalipes*, then Ovalipidae would be a valid family and species derived within Geryonidae would constitute a distinct genus, likely *Aeneacancer* Ward, 1933. Nevertheless, a new diagnosis of Geryonidae is provided below that incorporates Ovalipidae sensu Davie, Guinot & Ng (2015b).

Family Carcinidae MacLay, 1838

The second major well-supported portunoid clade recovered in this study consists of members from the portunoid families Carcinidae, Pirimelidae, Polybiidae, and Thiidae, as well as the inclusion of the caphyrine genus *Coelocarcinus* (Fig. 10; bs 64%, 93%, pp 1.0, 1.0). Here I propose that each of these lineages be recognized as a subfamily in the family Carcinidae (Fig. 5). A new diagnosis of Carcinidae is provided below. The composition and diagnoses of carcinid subfamilies will mostly follow that outlined (as families) by Spiridonov, Neretina & Schepetov (2014) and Davie, Guinot & Ng (2015b) but a more detailed treatment of the relationships within the family will be needed. For example, *Parathranites*’ position as the earliest diverging carcinid lineage renders Polybiinae polyphyletic (Fig. 10A). However, while *Parathranites* is morphologically distinct, the relatively low ML support in the backbone of the Carcinid topology suggests this placement is not robustly supported. Future efforts would benefit from analyses of more complete sequence data (i.e., less missing data) and greater taxon sampling (e.g., including more than one of the eight *Parathranites* spp.).

The novel placement of the Caphyrinae genus *Coelocarcinus* may be expected. These crabs are morphologically peculiar (Fig. 1B) and unlike most caphyrine crabs, they are not symbiotic—instead being found in association with *Halimeda*-sand, possibly mimicking dead segments of calcified algae (Ng, 2002; N. Evans, 2014, personal observation). Noting its peculiar morphology, Karasawa, Schweitzer & Feldmann (2008) proposed that *Coelocarcinus* belonged to the family Hepatidae Stimpson, 1871 (now Aethridae Dana, 1851). However, here I recover two *Coelocarcinus* taxa as a single long-branched clade within a well-supported Carcinidae. While phylogenetically long-branched taxa are more vulnerable to artifactual placement (Evans et al., 2010), additional analyses suggest that this was not the case for *Coelocarcinus*. ML analyses of the *Brusinia*-16S data set recovered the same placement for *Coelocarcinus* even though taxon sampling included hundreds of other brachyuran taxa (Fig. S6). Consequently, here I propose that Coelocarcininae Števčić (2005), is a valid carcinid subfamily.

Finally, concatenated analyses also recover *Polybius henslowii* as derived within a strongly supported *Liocarcinus* clade, as sister to *Liocarcinus holsatus* (Fig. 10). This result is consistent with previous molecular work (Plagge et al., 2016; Schubart & Reuschel, 2009; Spiridonov, Neretina & Schepetov, 2014), and given that *L. holsatus* and *P. henslowii* are generic types, the genera should be synonymized. However, while *Polybius* Leach, 1820, is the senior name, Plagge et al. (2016) proposed that the more widely used *Liocarcinus,* Stimpson, 1871, should take priority. Nevertheless, it is thought that a more detailed taxonomic revision will be needed and a final ruling by the ICZN may be prudent (Plagge et al., 2016; V. Spiridonov, 2017, personal communication).

Family Portunidae Rafinesque, 1815

The third well-supported major portunoid clade recovered here consists only of taxa belonging to Portunidae sensu Spiridonov, Neretina & Schepetov (2014), excepting *Coelocarcinus* (Figs. 10 and 11; bs 97%, 98%, pp 1.0, 1.0). These results confirm those of Schubart & Reuschel (2009) by recovering Portunidae as a distinct lineage that does not include carcinid crabs. Results regarding portunid subfamilies and genera are discussed in more detail below. For a diagnosis of the family see Davie, Guinot & Ng (2015b).

Family Brusiniidae Števčić, 1991

*Brusinia* is a morphologically peculiar genus of small, deep-sea crabs exhibiting many morphological features consistent with membership in Portunoidea (Fig. 1A). Originally assigned to the geryonid genus *Benthochascon*, this distinct lineage was raised to a generic rank by Števčić (1991) who also erected the tribe Brusiniini Števčić (1991). This clade was subsequently moved from Geryonidae to Carcininae (Crosnier & Moosa, 2002; Števčić, 2005), then to Polybiinae (Ng, Guinot & Davie, 2008; Karasawa, Schweitzer & Feldmann, 2008), and finally raised to family level status by Spiridonov, Neretina & Schepetov (2014). Nevertheless, some have noted that morphologically *Brusinia* remains an outlier in this family with “all male pleomeres free, somite 3 [lacking] a transverse keel, and the carapace [being] longer than wide” (Karasawa, Schweitzer & Feldmann, 2008). Here I generated the first molecular data for this genus consisting of 16S rRNA from *Brusinia profunda*. However, preliminary ML analyses failed to recover a placement of this species near or within Portunoidea and thus this sequence was left out of subsequent concatenated analyses. Consideration of lab procedures and extensive analyses of available Brachyura sequence data indicate that this sequence is not likely a contaminant so further analyses were conducted. ML analyses were carried out on *Brusinia profunda* in a data set comprised of 309 taxa using all 16S rRNA data from this study and all 16S data analyzed in Tsang et al. (2014). Results recovered *Brusinia* well outside a monophyletic Portunoidea (Fig. S6) albeit, with very low support. With some exceptions (and little to no support) the topology of Brachyura in this analysis was consistent with that recovered by Tsang et al. (2014) from a concatenated data set of eight genes. These results suggest that Brusiniidae may be a distinct lineage within the brachyuran subsection Heterotremata. However, further molecular and morphological work is needed to resolve the specific placement of this clade. For a diagnosis of the family see Davie, Guinot & Ng (2015b).

### Portunidae subfamilies

The validity and composition of portunid subfamilies have long been debated (reviewed in Davie, Guinot & Ng (2015a), Karasawa, Schweitzer & Feldmann (2008), Mantelatto et al. (2009), Nguyen (2013), Schubart & Reuschel (2009), Spiridonov, Neretina & Schepetov (2014)). There is consensus that most portunid subfamilies may not represent reciprocally monophyletic clades but are taxonomically useful groupings that should be retained until additional work is conducted (Davie, Guinot & Ng, 2015a). Chief among these, Portuninae and its largest genus *Portunus* are widely understood to be polyphyletic. However, Karasawa, Schweitzer & Feldmann (2008)—and to some extent Spiridonov, Neretina & Schepetov (2014)—departed from Portuninae *sensu* Ng, Guinot & Davie (2008) by recognizing the portunid subfamilies Atoportuninae, Lupocyclinae, Necronectinae, and Portuninae, in addition to the more generally accepted Caphyrinae, Carupinae, Podophthalminae, and Thalamitinae (Fig. 5). To the extent possible, the status of each of these portunid subfamilies is reevaluated here in light of the results of this study. However, while Thalamitinae and Caphyrinae are well sampled, it should be understood that most other portunid subfamilies are not. The greater phylogenetic resolution and higher support values recovered for Thalamitinae demonstrate that increased taxon sampling for other subfamilies should significantly improve future analyses of these groups. Yet results of this and other work also suggest that the molecular markers used here will likely never fully resolve deeper nodes in the family (e.g., see Lasley, Klaus & Ng, 2015; Thoma, Guinot & Felder, 2014).

Carupinae Paulson, 1875, sensu lato

Carupinae (Figs. 1C and 1D) is a fascinating group of morphologically peculiar, highly modified portunid crabs. Relative to other portunids members of this group are often smaller, smoother, with reduced eyes and much narrower natatory legs. Most attribute these modifications to their ecology as rubble-dwelling, cavernicolous, or even anchialine crabs (Fujita & Naruse, 2011; Ng, 2011; Ng & Takeda, 2003). This subfamily includes the genera *Carupa*, *Catoptrus*, *Kume*, *Libystes*, *Richerellus* and *Pele. Atoportunus* is also sometimes considered (Ng, 2011; Ng & Takeda, 2003); however, Karasawa, Schweitzer & Feldmann (2008) found morphological cladistic support for the subfamily Atoportuninae Števčić (2005), being comprised of *Atoportunus* and *Laleonectes*. Molecular phylogenetic work has subsequently supported an affinity of *Laleonectes* with Carupinae (Schubart & Reuschel, 2009; Spiridonov, Neretina & Schepetov, 2014). Together these findings led Spiridonov, Neretina & Schepetov (2014) to suggest that Carupinae sensu lato likely includes Atoportuninae. The present study includes the first molecular data generated for *Atoportunus*. Phylogenetic analyses of the 163 OTU concatenated data set recover a weakly supported monophyletic Carupinae + Atoportuninae clade (Fig. 11A; bs <50%, pp 1.0), but analyses of the 138 OTU data set do not (although they do not provide support against it; Fig. 11B). Consistent with previous molecular work (Schubart & Reuschel, 2009) and morphological discussions (Ng, 2011; Takeda, 2010), these analyses also recover *Carupa, Catoptrus* and *Lybistes* as poly- and paraphyletic. These findings include a placement of *Catoptrus nitidus* derived within or sister to *Lybistes* (Fig. 11; bs 99%, 100%, pp 1.0, 1.0). However, a second *Catoptrus* OTU (*Catoptrus* aff. *nitidus*) shared no affinity with *Lybistes*, instead grouping with *Atoportunus* (Fig. 11; bs 59%, 70%, pp <0.95, 0.98). These results should be approached with caution until more comprehensive molecular and morphological work are conducted on a well sampled Carupinae. Inclusion of *Kume* Naruse & Ng, 2012, and *Pele* Ng, 2011 may be particularly important given their close morphological affinity to *Lybistes* and *Catoptrus* (Naruse & Ng, 2012; Ng, 2011). Nevertheless, there is now some (though very weak) molecular support for a Carupinae sensu lato that includes *Atoportunus* and *Laleonectes*.

Lupocyclinae Paulson, 1875

Lupocyclinae sensu Karasawa, Schweitzer & Feldmann (2008) includes *Lupocyclus* and *Carupella*, while Lupocyclinae sensu Spiridonov, Neretina & Schepetov (2014) includes *Lupocyclus* and *Lupocycloporus*, but does not explicitly place *Carupella* anywhere. However, V. Spiridonov (2017, personal communication) has some doubt about the validity of *Carupella*, questioning whether it may instead represent juvenile specimens of one or more known portunine species (e.g., consider specimens examined by Vijaylaxmi, Padate & Rivonker (2016)). Data from *Carupella* was not available for analysis and here only weak support was recovered for a poorly sampled monophyletic Lupocyclinae (Fig. 11; bs <50%, <50%, pp <0.95, 0.99). The placement of *Lupocyloporus* renders *Lupocyclus* paraphyletic (Fig. 11). This is yet another fascinating, morphologically peculiar lineage of portunids that needs further work.

Necronectinae Glaessner, 1928

Necronectinae is comprised of the Indo-Pacific *Scylla* and monotypic West African *Sanquerus* Manning, 1989. The carapace of *Sanquerus* is similar to that of *Scylla*, but its chelipeds have a prismatic shape similar (at least superficially) to that of *Euphylax* (N. Evans, 2014, personal observation; e.g., see Manning, 1989). The present study did not include data for *Sanquerus* but analyzed all four *Scylla* species. Results recover strong support for the monophyly of *Scylla* (Fig. 11; bs 99%, 97%, pp 1.0, 1.0) with species relationships consistent to those recovered by Keenan, Davie & Mann (1998; based on CO1, 16S rRNA and allozyme data). *Scylla* demonstrates some phylogenetic affinity to *Podophthalmus* and Carupinae but this relationship exhibits no strong support. Additional analyses must include *Sanquerus*.

Podophthalminae Stimpson, 1860

This subfamily is comprised of the genera *Euphylax* and *Podophthalmus* (including *Vojmirophthalmus* Števčić, 2011 [=*Podophthalmus minabensis* Sakai, 1961]). These crabs exhibit unusually long eyestalks that render the orbital regions enormous and the frontal margin greatly reduced (Fig. 1F). The affinity of these genera has never been significantly challenged, but Garth & Stephenson (1966) noted significant differences between the morphology of the eyestalks, anterolateral carapace margin and G1s. Results presented here are the first to analyze the placement of these two genera together. Though data was limited for *Euphylax* (16S rRNA only), single marker and concatenated analyses failed to recover a monophyletic Podophthalminae (Fig. 11; Figs. S1 and S2). *Podophthalmus* demonstrated some topological affinity to Necronectinae and Carupinae, but always with little or no support. *Euphylax* showed no relative affinity to any portunid clade, instead always diverging alone from deeper nodes in Portunidae, but bearing no support. These results neither significantly challenge nor resolve the validity or composition of Podophthalminae.

Portuninae Rafinesque, 1815

As previously discussed, the monophyly of Portuninae and its largest genus, *Portunus* (98 extant species), has long been challenged. Some of this controversy can be attributed to an expansion of the genus by Stephenson & Campbell (1959) and Stephenson (1972), which included the incorporation of several morphologically similar but previously separate genera. Ng, Guinot & Davie (2008) mostly followed this classification, but retained many of these synonymized genera as subgenera (as did Sakai, 1976). A number of recent studies have provided evidence that these clades are morphologically and phylogenetically distinct, with some clearly worthy of generic status (Karasawa, Schweitzer & Feldmann, 2008; Mantelatto et al., 2009; Nguyen, 2013; Schubart & Reuschel, 2009; Spiridonov, Neretina & Schepetov, 2014). Consistent with these studies, phylogenetic analyses here recover a Portuninae comprised of at least three clades and a *Cronius* lineage (sensu Mantelatto et al., 2009) falling sister to Thalamitinae (Fig. 11; discussed below). The first of these clades, Portuninae sensu stricto (Clade I), is strongly supported and comprised of *Arenaeus, Callinectes* and some *Portunus* species, including the generic type *P. pelagicus* (Linnaeus, 1758) (Fig. 11; bs 96%, 97%, pp 1.0, 1.0). The second clade, Portuninae sensu lato Clade II, also exhibits significant support (Fig. 11; bs 86%, 88%, pp 1.0, 1.0) and is comprised mostly of *Portunus* (*Achelous)*, some *Portunus (Portunus)* and the monotypic *Lupella forceps*. Following Mantelatto et al. (2009) many have treated *Achelous* as a distinct but not fully revised genus (Spiridonov, Neretina & Schepetov, 2014; Nguyen, 2013). The third clade, Portuninae sensu lato “Clade” III, was weakly supported and comprised of the *Portunus* subgenera *Cycloachelous*, *Monomia* and a paraphyletic *Xiphonectes* (Fig. 11; bs 64%, 66%, pp <0.95, <0.95). Only the 174 OTU data set included multiple members of *Cycloachelous* and *Monomia* and analyses recovered strong support for the monophyly of *Monomia* (Fig. 13A; bs 74%, pp 1.0) but less support for the monophyly of *Cycloachelous* (Fig. 13A; bs <50%, pp 0.99). Finally, the 174 OTU analyses also recovered an unusual but poorly supported placement of *Portunus* (*Xiphonectes*) *tenuipes* within the portunid subfamily Thalamitinae (Fig. S1; bs <50%, pp <0.95). Using the same data for this species (CO1 and 313 bps of 28S rRNA) Spiridonov, Neretina & Schepetov (2014) discussed some concern when the same unusual placement was recovered. However, this result is likely artifactual and finds no other support from morphology or the molecular results presented here. Further work is clearly needed to resolve the systematics of *Portunus* sensu lato and Portuninae, neither of which were recovered here as monophyletic.

Thalamitinae Paulson, 1875

Following Stephenson (1972) Thalamitinae was placed in Portuninae where it stayed until Apel & Spiridonov (1998) reestablished the subfamily and provided a new morphological diagnosis of the group. Although Thalamitinae now represents the most diverse portunid subfamily (162 spp.; Spiridonov, Neretina & Schepetov, 2014; Spiridonov, 2017), many continue to question the validity of this group (Davie, Guinot & Ng, 2015a). This is partly attributable to the portunine genus *Cronius* (sensu Mantelatto et al., 2009) which exhibits a morphology intermediate to that of *Portunus* and the thalamitine genus *Charybdis* (Garth & Stephenson, 1966; Spiridonov, Neretina & Schepetov, 2014). This has suggested to some researchers that Thalamitinae may be derived within Portuninae. Lending credence to this, the molecular study of Mantelatto et al. (2009) recovered and discussed a derived clade comprised of the portunine genera *Cronius* + *Laleonectes* and a monophyletic Thalamitinae. However, results reported in Mantelatto et al. (2009) actually provide no significant support for this “clade,” with NJ and parsimony bootstrap values below 50% and a BI pp of 0.59. Conversely, while some have argued that *Cronius* may actually share a greater affinity with *Charybdis* than *Portunus* (Garth & Stephenson, 1966), only recently has it been suggested, based on morphological grounds, that *Cronius* might group with Thalamitinae rather than Portuninae (Spiridonov, Neretina & Schepetov, 2014). Results presented here support this view, recovering *Cronius* sister to Thalamitinae with little to moderate support (Figs. 11 and 12; bs <50%, 66%, pp <0.95, 1.0). Consequently, consideration of morphology (discussed below) and molecular data suggests that *Cronius* is more appropriately classified as a thalamitine crab. A new diagnosis of Thalamitinae is provided here which accommodates *Cronius*.

*Cronius* aside, results presented here also display strong support for a Thalamitinae that includes the Caphyrinae genera *Caphyra* and *Lissocarcinus* (Figs. 11 and 12; bs 68%, 92%, pp 0.97, 1.0). Furthermore, these two symbiotic genera also appear highly derived within an otherwise moderately supported *Thalamita* clade (Fig. 12; bs 62%, 66%, pp 1.0, 1.0). This result is not entirely novel given that the morphological affinity of Caphyrinae to Thalamitinae has long been recognized (Stephenson & Campbell, 1960), and its derived position has received some molecular support (Spiridonov, Neretina & Schepetov, 2014). However, results presented here represent the first comprehensive phylogenetic analyses of both subfamilies, including all described genera, and 70 of 162 Thalamitinae taxa and 12 of 26 Caphyrinae taxa (excluding *Coelocarcinus,* see above). Furthermore, while Caphyrinae’s placement renders both *Thalamita* and *Caphyra* paraphyletic, the derived monophyletic clade *Lissocarcinus* + *Caphyra* + *Thalamita* (=*Zygita*, gen. nov. and *Trierarchus*, gen. nov.) includes two *Thalamita* sensu lato species (=*Z. longifrons*, comb. nov. and *Z. murinae*, comb. nov.) recently demonstrated to be symbiotic (Evans & McKeon, 2016). Given the results of this work, Thalamitinae is redefined here to also include *Caphyra* and *Lissocarcinus*. For the sake of discussion, the *Lissocarcinus* + *Caphyra* + *Zygita* + *Trierarchus* clade is ascribed the subtribe name Caphyrina Paulson (1875), nomen translatum. Although the nature, degree, and phylogenetic pattern of symbiosis within Caphyrina clearly needs further study, this clade is dominated by commensal, symbiotic taxa (discussed below), which suggests a single origin of symbiosis for the group. Further highlighting the significance of this clade, symbiotic relationships have not been demonstrated in any other portunid taxa (but see the fascinating epibiotic ecology of *Portunus sayi* on floating *Sargassum* algae; Hartnoll, 1971; Russell & Dierssen, 2015; Turner & Rooker, 2006; West, 2012). One notable exception may be the numerous anecdotal observations of juvenile specimens of different portunoid species on gelatinous, nektonic organisms (V. Spiridonov, 2017, personal communication).

Finally, given the results of this study the following taxonomic changes are also discussed below: *Thalamonyx* A. Milne-Edwards, 1873, is reinstated as a valid genus; the subgenus *Goniosupradens* Leene, 1938, is raised to a generic rank; three new genera are described to accommodate some of the *Thalamita* sensu lato lineages rendered paraphyletic by Caphyrinae.

### Thalamitinae genera and subclades

Cronius *Stimpson, 1860*

Using 16S rRNA, Mantelatto et al. (2009) resurrected the species *Cronius edwardsii* (Fig. 8A), demonstrating that it was a genetically distinct geminate species of the generic type *Cronius ruber* (Fig. 2A). The same analyses also revealed that the remaining *Cronius* species, *Cronius timidulus*, is actually a member of *Achelous* [=*Portunus* (*Achelous*)]. These results are confirmed here with 16S rRNA and CO1 data from new specimens for all three species (Fig. 11).

Thalamitoides *A. Milne-Edwards, 1869*

*Thalamitoides* is a morphologically peculiar thalamitine genus with a short, laterally expanded carapace, exceptionally wide set eyes and a wide frontal margin (Fig. 2B). Though sometimes thought to have a greater affinity to *Thalamita*, phylogenetic results now place the genus sister to the remaining Thalamitinae, with moderate to strong support (Fig. 12; bs 68%, 92%, pp 0.97, 1.0).

Gonioinfradens *Leene, 1938*

Once classified as a subgenus of *Charybdis*, the monotypic *Gonioinfradens* (Fig. 2C) is easily distinguished from *Charybdis* by having four instead of six well-developed anterolateral teeth, and one to three subsidiary teeth (compare Figs. 8B–8D). The presence of such subsidiary anterolateral teeth occurs in only a few other *Charybdis* species. Leene (1938) recognized this morphology as distinct and to accommodate these species, described the *Charybdis* subgenera *Gonioinfradens* and *Goniosupradens*. More recently, *Gonioinfradens* (but not *Goniosupradens*) was raised to the generic rank (Apel & Spiridonov, 1998). Phylogenetic analyses presented here are the first to include either subgenus. Concatenated analyses recover *Gonioinfradens* as sister to a well-supported *Charybdis* sensu lato clade (including *Goniosupradens*). However, support for this placement is moderate or weak (Fig. 12; bs 59%, 56%, pp 1.0, <0.95).

Goniosupradens *Leene, 1938, status nov*.

Concatenated analyses recovered strong support for a reciprocally monophyletic clade including all three *Goniosupradens* species and *Charybdis hawaiensis* (Fig. 12; bs 99%, 100%, pp 1.0, 1.0). Moreover, this clade was strongly supported falling sister to a monophyletic *Charybdis* sensu stricto clade (Fig. 12; bs 97%, 99%, pp 1.0, 1.0). Although *Ch. hawaiensis* (=*Goniosupradens hawaiensis*, comb. nov.) was thought to be closely related to *Ch. orientalis* (Edmondson, 1954), a reevaluation of it morphology (discussed below) suggests that these similarities are superficial. Here *Goniosupradens* (Figs. 2D and 8C) is raised to the generic rank and a new diagnosis is provided that incorporates *G. hawaiensis*.

Charybdis *De Haan, 1833*

Concatenated analyses recovered a monophyletic *Charybdis* lineage (excluding *Goniosupradens*) with strong support (Fig. 12; bs 93%, 97%, pp 1.0, 1.0). There was no support for other proposed *Charybdis* subgenera (e.g., *Goniohellenus* and *Gonioneptunus*), although analyses included only 18 of 65 *Charybdis* species.

Thalamonyx *A. Milne-Edwards, 1873, status nov*.

The status of *Thalamonyx* has long been questioned as these crabs exhibit a peculiar morphology with similarities to *Thalamita*, *Charybdis* and *Caphyra* (Leene, 1938). This genus was synonymized with *Thalamita* by Stephenson & Hudson (1957). While this synonymy was widely accepted (Ng, Guinot & Davie, 2008) some continued to treat *Thalamonyx* as valid (Crosnier, 1962, 1984; Spiridonov, Neretina & Schepetov, 2014). Analyses presented here are the first to include molecular data for the genus and results recover strong support for *Thalamonyx gracilipes* falling sister to a *Thalamita* sensu stricto clade (Fig. 12; bs 90%, 96%, pp 0.99, 1.0). Given this taxon’s distinct morphology and that several *Thalamita* sensu lato clades will constitute additional genera (discussed below), the generic status of *Thalamonyx* is formally reinstated and a new diagnosis provided.

Thalamita *Latreille, 1829*

With 91 species, *Thalamita* is the largest portunid genus (Spiridonov, 2017). Unlike *Portunus*, the taxonomy of this group has been less controversial. However, *Thalamita* is morphologically diverse (sometimes confusingly so) and has always been thought to have a close affinity to *Charybdis*. Some have even suggested that the two genera may “constitute an unbroken series,” one blending into the other (Stephenson & Hudson, 1957). Results presented here do not support this view, instead recovering each genus in phylogenetically distinct clades. Nevertheless, the derived placement of Caphyrinae (=Caphyrina nom. trans.) within *Thalamita* renders this genus paraphyletic. With the exception of those *Thalamita* species falling within Caphyrina, three clades and one “grade” of *Thalamita* taxa were recovered. Each of these four clades are labeled in Fig. 12B and discussed below.

*Thalamita admete* (Herbst, 1803) is the generic type. With few exceptions members traditionally grouped with this species (e.g., see Stephenson & Hudson, 1957) are recovered here falling within a moderately supported *Thalamita* sensu stricto clade (Fig. 12; bs 57%, 64%, pp 0.99, 0.99). This clade includes only small to moderate sized *Thalamita* species that are morphologically similar and often hard to distinguish. They all exhibit two wide frontal lobes; often with equally wide, mostly parallel inner orbital margins (Figs. 8G and 8H). Male first gonopods (G1s) are long, less stout and never significantly flared relative to similarly sized *Thalamita* sensu lato taxa (compare Figs. 7A and 7B). Fourteen species were recovered in this clade, but the group likely includes many additional species not considered here. Nevertheless, some species traditionally assigned to this group were not recovered in the clade. Specifically, *Thalamita oculea* and *Th. sima* exhibit a similar size and carapace morphology to *Th. admete* but their gonopod morphology is different and phylogenetically they group with members of *Thalamita* sensu lato Clade III (discussed below). Unfortunately, at this time, developing a new diagnosis for *Thalamita* sensu stricto would be premature. While the present study does include over half of all *Thalamita* sensu lato taxa, several morphologically important species have not been included (e.g., *Th. annulipes*, *Th. margaritimana,* and *Th. platypenis*) and poor phylogenetic resolution at several critical nodes complicates the delineation of clades within the group. Additional work on *Thalamita* sensu lato is underway by V. Spiridonov and N. Evans both separately and in collaboration.

The remaining *Thalamita* sensu lato taxa and Caphyrina form a moderately well-supported clade (Fig. 12; bs 62%, 66%, pp 1.0, 1.0). In this clade the earliest diverging *Thalamita* sensu lato taxa form a grade (“Clade” II; Fig. 12) paraphyletic to the remaining *Thalamita* sensu lato clades (Clades III and IV). While carapace morphologies (e.g., frontal lobes and anterolateral teeth; Figs. 8I and 8J) vary substantially across this grade of small sized *Thalamita*, their G1s are diagnostically stout often with a laterally flared tip (Fig. 7B). However this G1 morphology is also shared among members of the *Thalamita* sensu lato Clade III. Clade III forms a distinct, strongly supported lineage (Fig. 12; bs 100%, 99%, pp 1.0, 1.0) of small to medium sized species. However, the diagnosis of this clade is complicated by some species having a two-lobed frontal margin striking similarity to that of *Thalamita* sensu stricto (Clade I), while others exhibit six frontal lobes similar to some members of *Thalamita* sensu lato “Clade” II (compare Figs. 8K with 8G and 8L with 8I). Finally, the monophyly of *Thalamita* sensu lato Clade IV is strongly supported (Fig. 12; bs 79%, 98%, pp 1.0, 1.0) and comprised only of large, morphologically similar *Thalamita* species. Given their morphology (discussed below) and the monophyly of this group here I establish the new genus *Thranita* to accommodate these species.

Caphyrina *Paulson, 1875, nom. trans*.

Here I recognize a moderately well supported clade (Fig. 12; bs 50%, 71%, pp 1.0, 1.0) comprised of *Caphyra*, *Lissocarcinus,* and six former *Thalamita* species as a redefined Caphyrina Paulson, 1875, nomen translatum (Figs. 3 and 9B–9J). Monophyly of *Lissocarcinus* was strongly supported (Fig. 12; bs 98%, 100%, pp 1.0, 1.0), and fell sister to a well-supported clade comprised of the remaining Caphyrina taxa (Fig. 12; bs 59%, 100%, pp 1.0, 1.0). This latter clade is morphologically diverse and includes a *Caphyra* sensu stricto clade as well as two lineages comprised of *Caphyra rotundifrons* and species formerly assigned to *Thalamita*. The first of these two lineages is comprised of the morphologically distinct geminate species *Th. longifrons* and *Th. murinae*. These species are both facultative commensals of nephtheid soft coral (Evans & McKeon, 2016) and have long been considered worthy of a generic status (Spiridonov & Neumann, 2008; Stephenson & Rees, 1967a). The new genus *Zygita* is described here to accommodate these species. The second lineage was recovered with poor support but includes *Thalamita woodmasoni* and *Thalamita cooperi*; species likewise considered part of a morphologically distinct *Thalamita* clade (the “Woodmasoni” group; Vannini, 1983). Although morphology strongly unites these taxa, phylogenetic results do not yet resolve whether they form a grade or a true clade within a well-supported Caphyrina. Though additional work is needed, here I establish the new genus *Trierarchus* comprised of these species (and all other “Woodmasoni” species) as well as *Thalamita squamosa* (=*Trierachus squamosus*, comb. nov.) and *C. rotundifrons* (=*Trierarchus rotundifrons*, comb. nov.). Limited but compelling data suggests that members of *Trierarchus* are symbiotic, forming facultative or obligate associations with algae (see Ecological remarks below). Furthermore, the placement of the morphologically divergent, obligate algal-commensal *C. rotundifrons* within *Trierarchus,* leaves a strongly supported *Caphyra* sensu stricto clade (Fig. 12; bs 100%, 100%, pp 1.0, 1.0). *Caphyra* now includes only species known to be commensal on soft corals. Finally, though analyses considered no more than seven of the 16 *Caphyra* sensu stricto species, results suggest that the genus may consist of two morphologically and ecologically distinct subclades; members of one clade (*Caphyra bedoti*, *Caphyra tridens*, and *Caphyra yookadai*) appear to primarily be obligate commensals of alcyoniid soft corals, where members of the other (including *Caphyra loevis* and *Caphyra* cf. *fulva*) are likely obligate commensals of xeniid soft corals (Crosnier, 1975b; Stephenson & Rees, 1968; collection data from UF holdings).

## Taxonomic Account

Portuniod family-level morphological diagnoses presented here amend those of Davie, Guinot & Ng (2015b), but do not address or impact diagnoses of Portunidae Rafinesque, 1815, or Brusiniidae Števčić (1991). New diagnoses are also presented for the portunid subfamily Thalamitinae and three new, and two revalidated thalamitine genera. Post-revision names are used for “Species included” lists, with junior synonyms in brackets “[ ].”

Superfamily Portunoidae Rafinesque, 1815

**Family Geryonidae Colosi, 1923**
Geryonidae Colosi, 1923: 249. Type genus *Geryon* Krøyer, 1837
Ovalipidae Spiridonov, Neretina & Schepetov (2014: 420). Type genus *Ovalipes* Rathbun, 1898

**Diagnosis:** Carapace ovate, hexagonal or subhexagonal, broader than long (sometimes only slightly so), smooth to moderately granular; regions variously expressed; epibranchial ridge of diffuse granules sometimes present, other carapace ridges not developed; frontal margin shorter than posterior margin, typically divided into even number of lobes or teeth (but sometimes three) with median notch present; supraorbital margin with one or two fissures, often indistinct (if two and distinct, central lobe toothed); infraorbital margin not separated from outer orbital lobe by fissure or notch; anterolateral margin with three to five teeth, shorter than posterolateral margin; posterolateral re-entrant not developed. Basal antennal segment free or fixed, longer than broad. Mesial lobe of first maxilliped endopod not well developed. Chelipeds heterochelous (sometimes weakly so) and heterodontous; merus typically without spines; carpus often with an outer spine; manus with dull, knob-like outer proximal spine. Meri of P2–P5 with antero-distal lobes or spines, sometimes reduced. P5 propodi laterally compressed, sometimes ovate; dactyli ovate, styliform, or lanceolate. Pleon with six somites plus telson typically distinguishable in both sexes with somites three to five in males separated by sutures but immovable. G1 long, opening anterolaterally. G2 thin, more or less approximately as long as G1. Diagnosis modified from Geryonidae and Ovalipidae sensu Spiridonov, Neretina & Schepetov (2014) and Davie, Guinot & Ng (2015b).

**Genera included:** *Benthochascon* Alcock & Anderson, 1899; *Chaceon* Manning & Holthuis, 1981; *Echinolatus* Davie & Crosnier, 2006; *Geryon* Krøyer, 1837; *Nectocarcinus* A. Milne-Edwards, 1861; *Ovalipes* Rathbun, 1898; *Raymanninus* Ng, 2000; *Zariquieyon* Manning & Holthuis, 1989.

**Remarks:** The placement of *Echinolatus* and *Nectocarcinus* within this family is tentative, following Davie, Guinot & Ng (2015b), but may be more appropriately judged “genera incertae sedis” considering remarks by Spiridonov, Neretina & Schepetov (2014). Additional morphological and phylogenetic work will be needed to resolve their placement.

**Family Carcinidae MacLeay, 1838**
Carcinidae MacLeay, 1838: 59. Type genus *Carcinus* Leach, 1814
Pirimelidae Alcock, 1899: 95. Type genus *Pirimela* Leach, 1814
Polybiidae Ortmann, 1893: 66. Type genus *Polybius* Leach, 1820
Thiidae Dana, 1852: 1425. Type genus *Thia* Leach, 1816

**Diagnosis:** Carapace hexagonal, subhexagonal, pyriform, or subcircular, rarely quasi-quadrate, typically broader than long; frontal margin sometimes entire, typically with two to four lobes or teeth, and shorter than posterior margin; inner supraorbital lobe weakly defined, significantly reduced, or absent; one or two supraorbital fissures sometimes reduced, rarely absent; anterolateral margin typically convex with five (sometimes four) teeth or lobes (count excluding exorbital tooth when small or poorly developed, e.g., as in some Pirimelinae); posterolateral re-entrant sometimes not developed, rarely absent. Basal antennal segment fixed, longer than wide. Well defined mesial lobe on endopod of first maxilliped sometimes present. Chelipeds typically heterochelous and heterodontous, sometimes symmetrical; merus typically without spines; carpus often with an outer spine; manus sometimes with dull, knob-like outer proximal spine; proximal inner surface of manus fixed finger concave. Meri of P2–P5 typically without antero-distal lobes or spines. P5 dactyli ovate (paddle-like), styliform, ensiform, or lanceolate. Sutures between sternites and episternites incomplete or partially incomplete. Pleonal somites three to five in males typically fused sometimes with traces of sutures, rarely all six somites and telson free (in Thiinae). G1 straight to slightly or distinctly curved, sometimes with spinules and soft setae. G2 distinctly shorter than G1. Vulva typically rounded or ovate, sometimes broader than long. Diagnosis modified from Carcinidae, Pirimelidae, Polybiidae, and Thiidae sensu Spiridonov, Neretina & Schepetov (2014) and Davie, Guinot & Ng (2015b), and including *Coelocarcinus* sensu Ng, 2002.

**Genera included:** *Bathynectes* Stimpson, 1871; *Carcinus* Leach, 1814; *Coelocarcinus* Edmondson, 1930; *Coenophthalmus* A. Milne-Edwards, 1873; *Liocarcinus* Stimpson, 1871; *Macropipus* Prestandrea, 1833; *Nautilocorystes* H. Milne-Edwards, 1837; *Necora* Holthuis, 1987; *Parathranites* Miers, 1886; *Pirimela* Leach, 1816; *Polybius* Leach, 1820; *Portumnus* Leach 1814; *Sirpus* Gordon, 1951; *Thia* Leach, 1816; *Xaiva* MacLeay, 1838.

Family Portunidae Rafinesque, 1815

**Subfamily Thalamitinae Paulson, 1875**
Figures 2–4, 6–9 and 14
Thalamitinae Paulson, 1875: 69. Type genus *Thalamita* Latreille, 1829
Caphyrinae Paulson, 1875: 69. Type genus *Caphyra* Guérin, 1832

**Figure 14:**
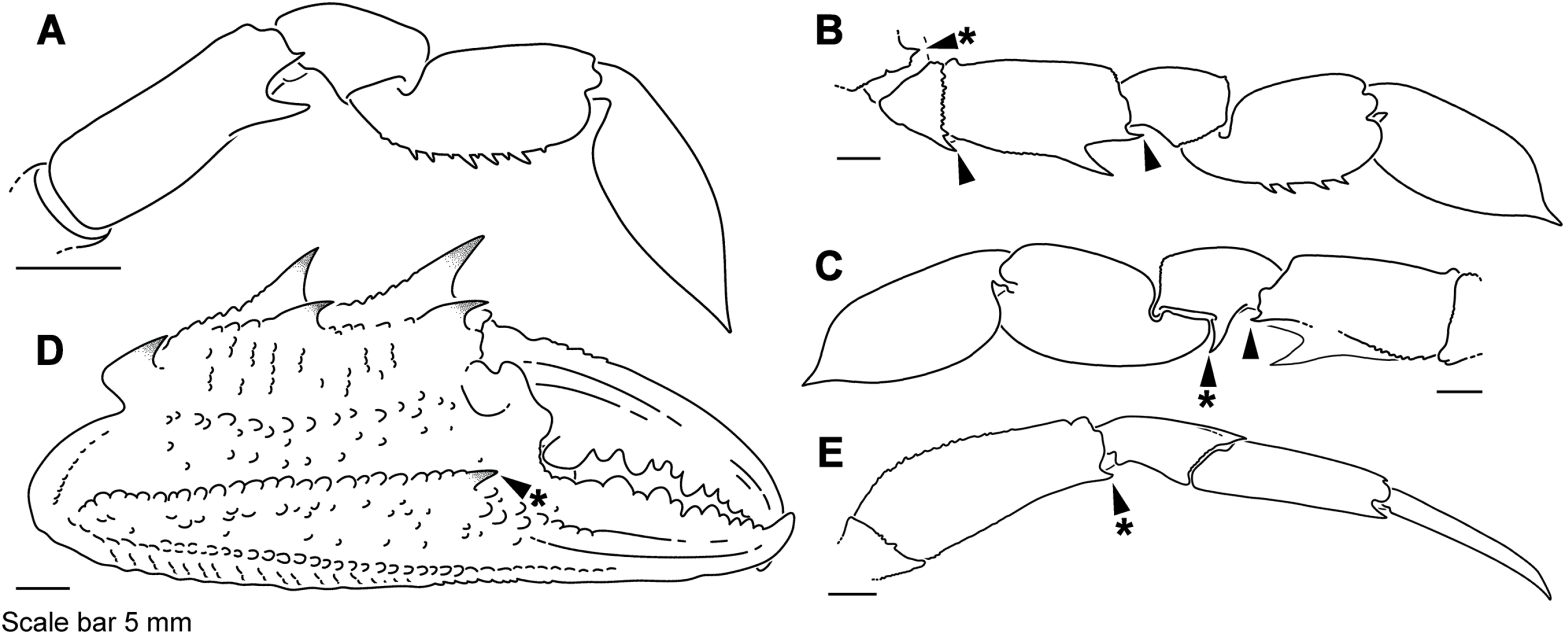
Relevant diagnostic morphology of *Trierarchus* gen. nov. and *Zygita* gen. nov. (A) Dorsal surface right P5, *Trierarchus cooperi* (UF 23802). (B–E) *Zygita longifrons* (UF 199): (B) dorsal surface right P5; (C) ventral surface right P5; (D) outer surface right cheliped; (E) right P4, ambulatory leg. Solid arrows, morphology discussed. Asterisks, important diagnostic traits.

**Diagnosis:** Carapace subcircular, subhexagonal or subtrapezoidal; slightly to substantially broader than long. Anterolateral margin with two to nine teeth, but typically four to six, and rarely nearly entire (e.g., some *Lissocarcinus* and *Caphyra*); if teeth number greater than six, five are typically large, well developed, and correspond to portunid teeth AT1, AT3, AT5, AT7 and AT9; the remaining being small, subsidiary, or rudimentary teeth appearing between the larger teeth (e.g., Figs. 8A–8C); rarely the first anterolateral tooth appears truncate and notched somewhat resembling a poorly developed additional tooth (e.g., *Charybdis feriata*). Basal antennal segment transversely broadened or lying obliquely, and entering or filling orbital hiatus; antennal peduncle and flagellum completely or nearly completely excluded from orbit (Fig. 6C). Chelipeds (P1) the same length or longer than ambulatory legs (P2–P4), typically bearing spines on the merus, carpus and manus; manus usually bearing one or more spines along both Carina 1 and Carina 2 and a well-developed outer proximal spine (Figs. 6D–6F; notable exceptions include many *Caphyra* and *Lissocarcinus* species). P5 typically with paddle-shaped propodi and dactyli, but sometimes otherwise modified (e.g., Figs. 4 and 14A). G1 with subdistal spinules, spines, bristles, or “hairs.” Diagnosis modified from Thalamitinae and Caphyrinae sensu Apel & Spiridonov (1998), *Cronius* sensu Garth & Stephenson (1966), and *Caphyra* sensu Apel & Steudel (2001).

**Genera included:** *Caphyra* Guérin, 1832; *Charybdis* De Haan, 1833; *Cronius* Stimpson, 1860; *Gonioinfradens* Leene, 1938; *Goniosupradens* Leene, 1938, status nov.; *Lissocarcinus* Adams & White, 1849; *Thalamita* Latreille, 1829; *Thalamitoides* A. Milne-Edwards, 1869; *Thalamonyx* A. Milne-Edwards, 1873, status nov.; *Thranita*, gen. nov.; *Trierarchus*, gen. nov.; *Zygita*, gen. nov.

**Remarks:** With the addition of *Caphyra, Cronius* and *Lissocarcinus*, Thalamitinae now includes 190 species (Spiridonov, Neretina & Schepetov, 2014; Spiridonov, 2017) and is the largest portunoid subfamily. *Cronius* notably expands the diagnosis of the subfamily to include two species with nine anterolateral teeth. However, *Cronius* clearly exhibits morphology diagnostic of Thalamitinae including exclusion of the antennal flagellum from the orbit by the basal antennal joint, no more than six large anterolateral teeth, and a G1 with subterminal bristles or “hairs” (Garth & Stephenson, 1966; Spiridonov, Neretina & Schepetov, 2014).

**Genus *Goniosupradens*Leene, 1938, status nov.**
Figures 2D, 4A and 8C

**Type species:** *Portunus erythrodactylus* Lamark, 1818, subsequent designation by Davie (2002); gender masculine.

**Diagnosis:** Carapace subhexagonal, slightly broader than long. Frontal margin with six well-developed teeth or lobes of approximately equal width. Anterolateral margin with five large, well-developed, forward-sweeping teeth corresponding to portunid teeth AT1, AT3, AT5, AT7 and AT9 and one or two subsidiary teeth corresponding to teeth AT2 (always present) and AT4 (sometimes present); subsidiary teeth not significantly swept forward but terminating approximately perpendicular to the anterolateral margin; epibranchial tooth (AT9) subequal to and never significantly extending laterally beyond tooth AT7. Posterior margin of carapace forming a curve with the posterolateral margin. Cheliped carinae 3–5 always well developed. Diagnosis modified from Leene (1938) to include *G. hawaiensis*, comb. nov., after Edmondson (1954).

**Species included:** *Goniosupradens acutifrons* (De Man, 1879); *Goniosupradens erythrodactylus* (Lamarck, 1818) [=*Thalamita pulchra* Randall, 1840; *Thalamita teschoiraei* A. Milne-Edwards, 1859]; *G. hawaiensis* (Edmondson, 1954), comb. nov.; *Goniosupradens obtusifrons* (Leene, 1937).

**Remarks:** Historically, *G. hawaiensis* (=*Charybdis hawaiensis*) was considered closely related to *Ch. orientalis* (Edmondson, 1954), but similarities are superficial. Once thought diagnostic of these species, the first “subsidiary” anterolateral tooth (AT2) is more reduced in *G. hawaiensis* in a manner consistent with other *Goniosupradens*. Furthermore, *G. hawaiensis*, like all *Goniosupradens*, bears an epibranchial, anterolateral tooth (AT9) subequal to, but never significantly extending laterally beyond the preceding tooth (AT7). The opposite condition is present in *Ch. orientalis* and most *Charybdis* (compare Figs. 8C and 8D).

**Genus *Thalamonyx* A. Milne-Edwards, 1873, status nov.**
Figures 2F, 7G and 8F

**Type species:** *Goniosoma danae* A. Milne-Edwards, 1869, subsequent designation by Rathbun, 1922; gender masculine.

**Diagnosis:** Carapace subhexagonal, approaching subcircular; moderately convex dorsally; mature specimens always slightly broader than long. Frontal margin of carapace not much wider than posterior margin and comprised of two lobes separated by a small, distinct notch and extending forward well beyond the inner supraorbital margin; lobes frequently slightly sinuous or concave near the inner margin such that each appears subtly bilobed. Inner supraorbital margin arched and less than one third as wide as frontal lobes. Five, sharp anterolateral teeth (AT1, AT3, AT5, AT7, and AT9); AT1 largest and directed forward; remaining subequal and swept forward forming an oblique, inclined border similar to that in *Charybdis*. Chelipeds subequal, not robust, and lightly granular all over; posterior border of merus subtly squamous; manus with one spine present along Carina 1, one spine along Carina 2, and a well-developed outer proximal spine; Carina 3–5 granular but increasingly well developed; Carina 6 granular or smooth and poorly developed; Carina 7 sometimes granular and well developed. Posterior border of P5 propodi without spinules. G1 short, stout, curved, broadening slightly toward a obliquely ending tip; subdistal lateral margin bearing stout, mostly paired bristles numbering approximately nine, preceded by additional thinner bristles; subdistal mesial margin with approximately five long, hook-shaped bristles followed by approximately four mostly straight, variously angled bristles. Female genital opening relatively large, located near anterior margin of sternite.

**Species included:** *Thalamonyx danae* (A. Milne-Edwards, 1869) [=*Thalamita anomala* Stephenson & Hudson, 1957]; *Th. gracilipes* A. Milne-Edwards, 1869.

**Remarks:** G1 morphology of this genus is diagnostic (Fig. 7G; see also Stephenson & Rees, 1967b, Fig. 2D; Nguyen, 2013, Fig. 15), as is the female genital opening (pers. comm. V. Spiridonov, 2017). Ng, Guinot & Davie (2008, Note 25, p. 158) created some confusion by misidentifying the type species of this genus which is *G. danae* A. Milne-Edwards, 1869, not *Th. danae* Stimpson, 1858. When Stephenson & Hudson (1957) designated *Thalamonyx* a junior synonym of *Thalamita*, the species *Th. danae* (A. Milne-Edwards, 1869) became a secondary homonym of *Th. danae* Stimpson, 1858. Consequently, *Th. danae* (A. Milne-Edwards, 1869) was given the new specific epithet *Th. anomala* (Stephenson & Hudson, 1957). In accordance with Article 59.4 of ICZN (1999), *Th. danae* (A. Milne-Edwards, 1869) is reinstated here as a valid species and *Th. anomala* (Stephenson & Hudson, 1957) its junior synonym. Though no *Th. danae* (A. Milne-Edwards, 1869) specimens were examined for this study, its sister status with *Th. gracilipes* (sampled here) is without question. The synonymization of these species has been discussed, but has never been fully investigated or formally adopted (discussed in Stephenson & Rees, 1967b). Finally, as others have noted, the *Thalamonyx* specimen illustrated by Crosnier (1962, Fig. 153) is that of an immature male; both its carapace and G1 are not fully developed and should not be used to diagnose adult specimens.

**Genus *Thranita*, gen. nov.**
Figures 2I, 4A, 7C and 9A
urn:lsid:zoobank.org:act:8122DA1E-5C68-45E2-91B0-76583068FF80

**Type species:** *Thalamita crenata* Rüppell, 1830, by present designation; gender feminine.

**Diagnosis:** Carapace subhexagonal, always broader than long, somewhat flattened and never significantly convex; frontal margin with six well-developed, bluntly rounded lobes of approximately even width; anterolateral margin with five (rarely four) well-developed sharp teeth corresponding to portunid teeth AT1, AT3, AT5, AT7, and AT9 (Fig. 9A); exorbital tooth (AT1) always entire; AT7 sometimes reduced or absent (e.g., in *Thranita pseudopelsarti*). Basal antennal segment always transversely broadened, never laying significantly oblique to orbital hiatus. G1 long, slightly tapering (Fig. 7C), rarely with a laterally recurved tip (e.g., as in *Thranita foresti*); never stout with a laterally flared tip.

**Species included:** *Thranita cerasma* (Wee & Ng, 1995), comb. nov. [=*Thalamita cerasma rectifrons* Crosnier & Moosa, 2002]; *Thranita coeruleipes* (Hombron & Jacquinot, 1846), comb. nov.; *Thranita crenata* (Rüppell, 1830), comb. nov.; *Thranita danae* (Stimpson, 1858), comb. nov. [*=Thalamita stimpsoni* A. Milne-Edwards, 1861; *Thalamita prymna* var. *proxima* Montgomery, 1931]; *Th. foresti* (Crosnier, 1962), comb. nov. [=?*Thalamita helleri* Hoffmann, 1874]; *Thranita gurjanovae* (Tien, 1969), comb. nov.; *Thranita holthuisi* (Stephenson, 1975), comb. nov.; *Thranita kotoensis* (Tien, 1969), comb. nov.; *Thranita pelsarti* (Montgomery, 1931), comb. nov.; *Thranita prymna* (Herbst, 1803), comb. nov. [*=Thalamita crassimana* Dana, 1852;* Thalamita pyrmna* var. *annectans* Laurie, 1906];* Thranita pseudopelsarti* (Crosnier, 2002), comb. nov.; *Thranita rubridens* (Apel & Spiridonov, 1998), comb. nov.; *Thranita spinicarpa* (Wee & Ng, 1995), comb. nov.; *Thranita spinimana* (Dana, 1852), comb. nov.; *Thranita starobogatovi* (Tien, 1969), comb. nov.; *Thranita tenuipes* (Borradaile, 1902), comb. nov.; *Thranita williami* (Spiridonov, 2017), comb. nov.

**Remarks:** Sometimes referred to as the “Prymna” group (after *Thalamita prymna*; Stephenson & Hudson, 1957), members of this clade include nearly all large species of *Thalamita* sensu lato and have long been recognized as morphologically similar. Though several of these species were not available (or suitable) for molecular analyses (e.g., *Th. cerasma*, *Th. pelsarti*, and *Th. williami*), they have always been considered morphologically most similar to species that were included here (e.g., Wee & Ng, 1995; Spiridonov, 2017). Although there is no striking single synapomorpy for *Thranita*, all members share a similar shaped carapace with six frontal lobes of approximately uniform shape and width (Fig. 9A) and a relatively long, gradually tapering G1 (Fig. 7C). This combination of characters is not seen in any other *Thalamita* clade.

**Etymology:** *Thalamita* Latreille, 1829 (and its suppressed, objective synonym *Thalamites* Guérin, 1829, a *nomen oblitum*; Low, Ng & Evenhuis, 2013) was named after Thalamite, the title given to oarsmen occupying the lowest tier of a trireme (a three-tiered ancient Greek warship). Keeping with this tradition, *Thranita* originates from Thranite, the title given to oarsmen occupying the upper tier of a trireme. Gender feminine.

**Genus *Trierarchus*, gen. nov.**
Figures 3F–3J, 4F, 7E, 7F, 9F–9J and 14A
urn:lsid:zoobank.org:act:5E5BD1B5-524D-46C4-9FFD-EA5FBF1C5239

**Type species:** *Thalamita woodmasoni* Alcock, 1899, by present designation; gender masculine.

**Diagnosis:** Carapace subhexagonal to subcircular, typically broader than long and somewhat convex; frontal margin flat or rounded and comprised of one to six (typically four) weakly distinguished lobes; four lobed specimens typically with median lobes approximately three times the width of lateral lobes; inner supraorbital margin sometimes nearly absent (e.g. *Tr. rotundifrons)* but typically subtly rounded and oblique with a breadth never greater than one-third the total breadth of the frontal lobes; anterolateral margin not reduced nor exhibiting a significantly concave epibranchial ridge (e.g., as in some *Caphyra*; Fig. 9E); anterolateral margin with four well-developed teeth swept forward corresponding to portunid teeth AT1, AT3, AT5 and AT9; a rudimentary tooth AT7 sometimes present (Figs. 9F–9J). Carapace and chelipeds subtly to substantially granular and covered with plumose setae. Chelipeds with posterior surface of merus bearing distinct granular squamiform markings; manus with weakly squamiform markings extending ventrally from Carina 5 to a poorly defined Carina 6. P5 dactyli typically lancelet-shaped, especially in juveniles, but approaching paddle-shaped in larger specimens (Figs. 14A and 3F, respectively). G1 curved and swelling slightly toward a club-shaped end with a bluntly rounded tip; subterminal bristles always present on abdominal-mesial surface, typically dense and comprised of several rows extending sparsely or densely to the sternal surface, merging with bristles of the lateral abdominal surface that extend to tip (Figs. 7E and 7F); larger subterminal bristle sockets distinct and often visible when bristles are damaged or missing.

**Species included:** *Trierarchus acanthophallus* (Chen & Yang, 2008), comb. nov.; *Trierarchus cooperi* (Borradaile, 1902), comb. nov.; *Trierarchus corrugatus* (Stephenson & Rees, 1961), comb. nov.; *Trierarchus crosnieri* (Vannini, 1983), comb. nov.; *Trierarchus demani* (Nobili, 1905), comb. nov. [=?*Thalamita trilineata* Stephenson & Hudson, 1957; ?*Thalamita invicta* Thallwitz, 1891]; *Trierarchus hanseni* (Alcock, 1899), comb. nov.; *Trierarchus procorrugatus* (Dai et al., 1986), comb. nov.; *Trierarchus quadridentatus* (Dai, Cai & Yang, 1996), comb. nov.; *Trierarchus rotundifrons* (A. Milne-Edwards, 1869), comb. nov.; *Trierarchus sankarankuttyi* (Crosnier & Thomassin, 1974), comb. nov.; *Trierarchus squamosus* (Stephenson & Hudson, 1957), comb. nov.; *Trierarchus woodmasoni* (Alcock, 1899), comb. nov.; *Trierarchus taprobanicus* (Alcock, 1899), comb. nov.

**Remarks:** The most diagnostic morphology of *Trierarchus* includes the G1, anterolateral margin and presence of squamiform markings and plumose setae. The G1 can be particularly useful (e.g., see also Crosnier, 1975a, Fig. 8; Crosnier & Thomassin, 1974, Fig. 8D; Chen & Yang, 2008, Fig. 7; Dai et al., 1986, Fig. 137A), however, both *Tr. squamosus* and *Tr. rotundifrons* possess divergent G1s (Stephenson & Hudson, 1957, Figs. 2K and 3K; Stephenson & Campbell, 1960, Figs. 1H and 2J). Additionally, *Tr. rotundifrons* is overall morphologically highly divergent from other members of this genus, likely due to its ecology as an obligate commensal. This species is smooth and lacks squamiform markings, most plumose setae, and most carinae on the chelipeds. Furthermore, its P5 dactyli are highly modified for firmly grasping host algae (Fig. 4F). Nevertheless, *Tr. rotundifrons* clearly displays a morphological affinity with other *Trierarchus*, most notably *Tr. woodmasoni* (compare Figs. 3F, 3I, 9F and 9G). Likewise, while *Tr. rotundifrons* was originally described in *Camptonyx* Heller, 1861, (an available junior synonym of *Caphyra*) it shares no close morphological or ecological affinity to *C. polita* (Heller, 1861), the type species of this invalid genus. *Caphyra polita* is a soft coral commensal with close morphological affinity to *C. fulva* (sampled here) and other *Caphyra* sensu stricto taxa (Crosnier, 1975b). Finally, it is worth noting that species delineations within *Trierarchus* remain problematic and a revision of this new genus is needed (e.g., see Crosnier, 1975a). Morphologically *Tr. sankarankuttyi* and *Tr. procorrugatus* have a strong affinity with *Tr. cooperi*, but they were described from limited material and interspecific differences were inadequately addressed (see Crosnier & Thomassin, 1974; Dai et al., 1986). Furthermore, while two well supported, genetically distinct *Tr*. cf. *cooperi* lineages were recovered here (sp. A and sp. B; Figs. 3G and 3H), examination of multiple DNA barcoded specimens from each lineage failed to reveal clear morphological distinctions between the taxa (from preliminary analyses with unpublished data; but see discussion of color below). Moreover, many individuals from both OTUs fit a diagnosis of *Tr. corrugatus* (Stephenson & Rees, 1961). This inter- and intraspecific variation likely explains why Crosnier (1962) synonymized this species with *Tr. cooperi*, though they are currently treated as distinct (Ng, Guinot & Davie, 2008; Nguyen, 2013). Comparison of sequenced specimens of *Tr. woodmasoni* from across the Indo-Pacific (from preliminary analyses with unpublished data) also suggests that *Tr. crosnieri, Tr. taprobanicus,* and *Tr. woodmasoni* may be intraspecific variants. Thus, *Trierarchus* is likely comprised of fewer valid species than currently recognized, but more detailed studies will be needed.

**Ecology:** Members of *Trierarchus* typically inhabit high-energy, shallow marine environments, often in association with algae (Vannini, 1983; Hay et al., 1989; UF collection data; personal observations). In Guam *Tr. rotundifrons* is always found in association with *Chlorodesmis* algae in exposed reefs, *Tr*. cf. *cooperi* is recovered by sieving living *Halimeda* (note light green live color in sp. B; Fig. 3H), and *Tr. woodmasoni* is reliably recovered from sieving *Sargassum* and other co-distributed algae. Around Moorea Island, French Polynesia, *Tr*. cf. *cooperi* is typically recovered by sieving and breaking coral rubble from fore reef environments. However, unlike *Tr*. cf. *cooperi* recovered in Guam, the species collected in Moorea (sp. A; Fig. 3G) displays a live color mottled with red, orange and purple hues—shades common among coralline algae, sponges and other encrusting marine life in such substrate. Nevertheless, with the exception of *Tr. rotundifrons*, which is demonstrably an obligate commensal (Hay et al., 1989), other symbiotic associations suggested for this genus remain speculative and need further study. Finally, in contrast to other species, the rarely collected *Tr. squamosus* appears to prefer protected lagoonal waters, but no further microhabitat or live color data are available for the species.

**Etymology:** A trierach (Latin *trierachus*) is the captain of a trireme, an ancient Greek warship. For context see Etymology of *Thranita* (above). Gender masculine.

**Genus *Zygita*, gen. nov.**
Figures 3E, 7D, 9C, 14B–14E
urn:lsid:zoobank.org:act:5CED217C-3FD0-4090-973D-529226942966

**Type species:** *Goniosoma longifrons* A. Milne-Edwards, 1869, by present designation; gender feminine.

**Diagnosis:** Carapace subhexagonal, approximately 1.5 times broader than long; frontal margin with six well-developed teeth of approximately even width separated by deep notches; inner supraorbital margin oblique and spiniform; anterolateral margin with five large, well-developed sharp teeth forming an oblique, inclined border reminiscent of *Thalamonyx* and *Charybdis* (Fig. 9C). Infraorbital lobe well developed and terminating in a spiniform or blunt point. Carapace, chelipeds and ambulatory legs subtly to substantially granular and covered with plumose setae (easily worn away in preserved specimens). Cheliped meri with a ventral anterodistal spine; carpus with additional dorsal spine between the typical three outer spines and a well-developed inner spine; manus Carina 4 distinct, granular and ending distally in a sharp or dull spinule (Fig. 14D), squamiform sculpture extending ventrally from Carina 5 to a poorly defined Carina 6. Meri of P2–P4 bearing a ventral posterodistal spine (Fig. 14E). P5 coxae bearing a stout, well-developed spinule dorsad; ischia with granular to spiniform distal border; meri with both a dorsal and ventral posterodistal spine; carpi with a well-developed spine on ventral posterodistal border; dactyli lancelet-shaped (especially in juveniles), but approaching paddle-shaped in larger individuals (Figs. 14B and 14C). G1 curved and tapering with a row of 1–12 subterminal bristles on lateral margin beginning just behind the tip; bristles continue sparsely across sternal surface extending to the mesial margin; mesial margin with similar, sometimes numerous spines beginning immediately behind tip (Fig. 7D).

**Species included:** *Zygita longifrons* (A. Milne-Edwards, 1869), comb. nov. [=*Thalamita spinimera* Stephenson & Rees, 1967; *Thalamita yoronensis* Sakai, 1969]; *Zygita murinae* (Zarenkov, 1971), comb. nov.

**Remarks:** The distinct morphology of this rarely collected genus is well known and deserving of generic rank (Stephenson & Rees, 1967a; Spiridonov & Neumann, 2008). The most diagnostic traits include the presence of a sharp or dull spinule at the distal end of manus Carina 4; meri of the ambulatory legs bearing a ventral posterodistal spine (Fig. 14E; asterisked); P5 coxa bearing a stout, well-developed dorsal spinule (Fig. 14B; asterisked); P5 carpus with a well-developed spine on the ventral posterodistal border (Fig. 14C; asterisked).

**Ecology:** In their original description of *Thalamita spinimera,* Stephenson & Rees (1967a) suggested these crabs were “ectocommensal” on Alcyonaria (=Octocorallia). However, this was based on one specimen collected from soft coral. A subsequent revision of this group by Spiridonov & Neumann (2008) failed to confirm this association, but only considered seven specimens. Evans & McKeon (2016) compiled compelling in situ photographs and collections records for 24 specimens and found that 46% (11 specimens) were found in association with soft corals (seven on nephtheid soft corals) in what is likely a facultative association.

**Etymology:** *Zygita* originates from Zygite, the title given to oarsmen occupying the middle tier of a trireme (a three-tiered ancient Greek warship). For context see Etymology of *Thranita* (above). Gender feminine.

## Conclusion

This study constitutes the most comprehensive molecular phylogenetic analyses of Portunoidea to date, but highlights numerous areas where additional work is needed. Results support a more conservative classification of Portunoidea with three instead of eight extant families: Geryonidae (Geryonidae + Ovalipidae; new diagnosis provided), Carcinidae (Carcinidae + Pirimelidae + Polybiidae + Thiidae + *Coelocarcinus*; new diagnosis provided) and Portunidae. Limited molecular data also suggest that the family Brusiniidae may still be valid, but might not be a portunoid lineage. A major aim of this study was to investigate the molecular phylogenetic origin of symbiosis within Portunoidea by substantially increasing taxon sampling of the subfamilies Caphyrinae and Thalamitinae. Results support a shared ancestry of all symbiotic taxa (*Caphyra*, *Lissocarcinus,* and two *Thalamita*) derived within the thalamitine genus *Thalamita*. Consequently, Caphyrina Paulson, 1875, nom. trans., should be considered a subtribe within the subfamily Thalamitinae. Although the nature, degree, and phylogenetic pattern of symbiosis within Caphyrina needs further study, this clade is clearly dominated by symbiotic taxa and likely originated from a symbiotic ancestor. Results presented here also support the following taxonomic actions within Thalamitinae: *Cronius* is reclassified as a thalamitine rather than a portunine genus; *Thalamonyx* is reinstated as a valid genus; *Goniosupradens* is raised to the generic rank; and three new genera (*Zygita* gen. nov., *Thranita* gen. nov., and *Trierarchus* gen. nov.) are described to accommodate some *Thalamita* sensu lato taxa rendered paraphyletic by Caphyrina. A new diagnosis of Thalamitinae has also been provided.

## Supplemental Information

10.7717/peerj.4260/supp-1 Supplemental Information 1 ML phylogram of Portunoidea based on analyses of 174 OTUs and a 3313 bp alignment of partial 16S rRNA, CO1, 28S rRNA, and H3 sequence data. Support values appear below relevant branches with ML bootstrap values ≥50% (based on 500 replicates) appearing first followed by BI posterior probabilities ≥0.95.

10.7717/peerj.4260/supp-2 Supplemental Information 2 ML phylogram of Portunoidea based analyses of 163 taxa and an 1105 bp alignment of partial mitochondrial 16S rRNA (+tRNA-Leu and NADH1) sequence data. Support values (%) appear above each relevant node and are based on 500 bootstrap replicate ML searches.

10.7717/peerj.4260/supp-3 Supplemental Information 3 ML phylogram of Portunoidea based analyses of 148 taxa and an 657 bp alignment of partial mitochondrial CO1 sequence data. Support values (%) appear above each relevant node and are based on 500 bootstrap replicate ML searches.

10.7717/peerj.4260/supp-4 Supplemental Information 4 ML phylogram of Portunoidea based analyses of 66 taxa and an 1224 bp alignment of partial 28S rRNA sequence data. Support values (%) appear above each relevant node and are based on 500 bootstrap replicate ML searches.

10.7717/peerj.4260/supp-5 Supplemental Information 5 ML phylogram of Portunoidea based analyses of 123 taxa and an 327 bp alignment of partial H3 sequence data. Support values (%) appear above each relevant node and are based on 500 bootstrap replicate ML searches.

10.7717/peerj.4260/supp-6 Supplemental Information 6 ML phylogram of *Brusinia profunda* and 308 mostly brachyuran taxa based on analyses of a 447 bp alignment of 16S rRNA sequence data. Support values (%) appear above each relevant node and are based on 500 bootstrap replicate ML searches. Brusiniidae and Portunoidea are highlighted.

10.7717/peerj.4260/supp-7 Supplemental Information 7 Expanded details regarding taxon sampling, GenBank accession numbers, and operational taxonomic unit composition of sequence data used for phylogenetic analyses.

10.7717/peerj.4260/supp-8 Supplemental Information 8 Best scoring partition schemes for four single marker molecular data sets.

## Additional Information and Declarations

### DNA Deposition

The following information was supplied regarding the deposition of DNA sequences: GenBank Accession numbers are provided in Table 1 and again in Table S1. Additional sequences discussed in text (as Genbank numbers) but not displayed in Tables were KT365713, KT365786, KT588224, and KX425018.

### Data Availability

The following information was supplied regarding data availability: Sequence alignments and phylogenetic results were deposited to TreeBASE and are accessible at https://treebase.org/treebase-web/search/study/summary.html?id=21486.

### New Species Registration

The following information was supplied regarding the registration of a newly described species: Publication LSID: urn:lsid:zoobank.org:pub:90E97894-9BBE-452C-A6A8-AFF7C1B78874; Genus Thranita gen. nov.: urn:lsid:zoobank.org:act:8122DA1E-5C68-45E2-91B0-76583068FF80; Genus Trierarchus gen. nov.: urn:lsid:zoobank.org:act:5E5BD1B5-524D-46C4-9FFD-EA5FBF1C5239; Genus Zygita gen. nov.: urn:lsid:zoobank.org:act:5CED217C-3FD0-4090-973D-529226942966.
