# Supplementary figures and images for: Molecular phylogenetics of swimming crabs (Portunoidea Rafinesque, 1815) supports a revised family-level classification and suggests a single derived origin of symbiotic taxa
Source: PeerJ. 2018 Jan 23;6:e4260. doi: 10.7717/peerj.4260 (PMC5786103; doi:10.7717/peerj.4260)

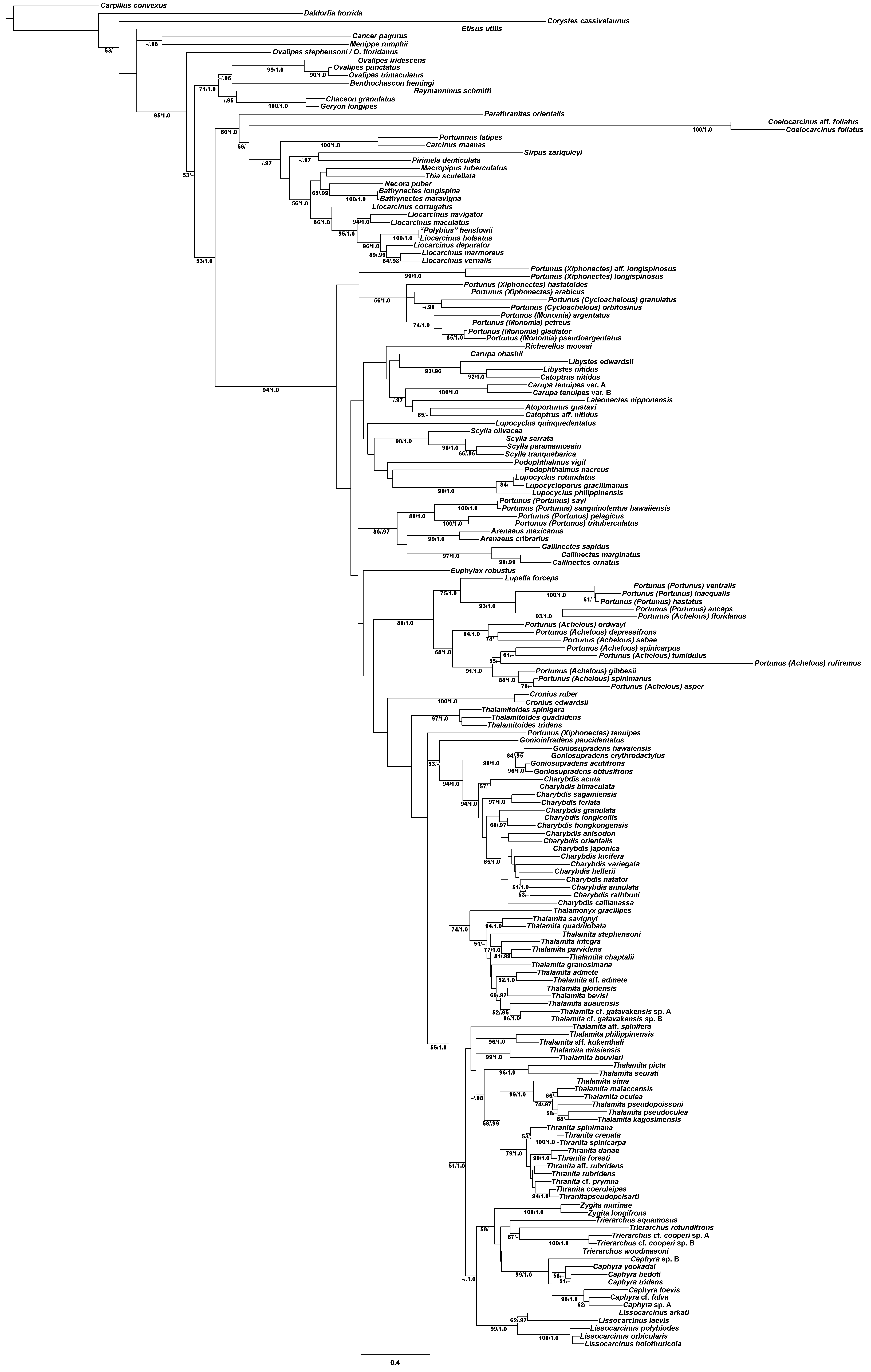

Supplement: Supplemental Information 1 — Support values appear below relevant branches with ML bootstrap values ≥50% (based on 500 replicates) appearing first followed by BI posterior probabilities ≥0.95. [file peerj-06-4260-s001.png]

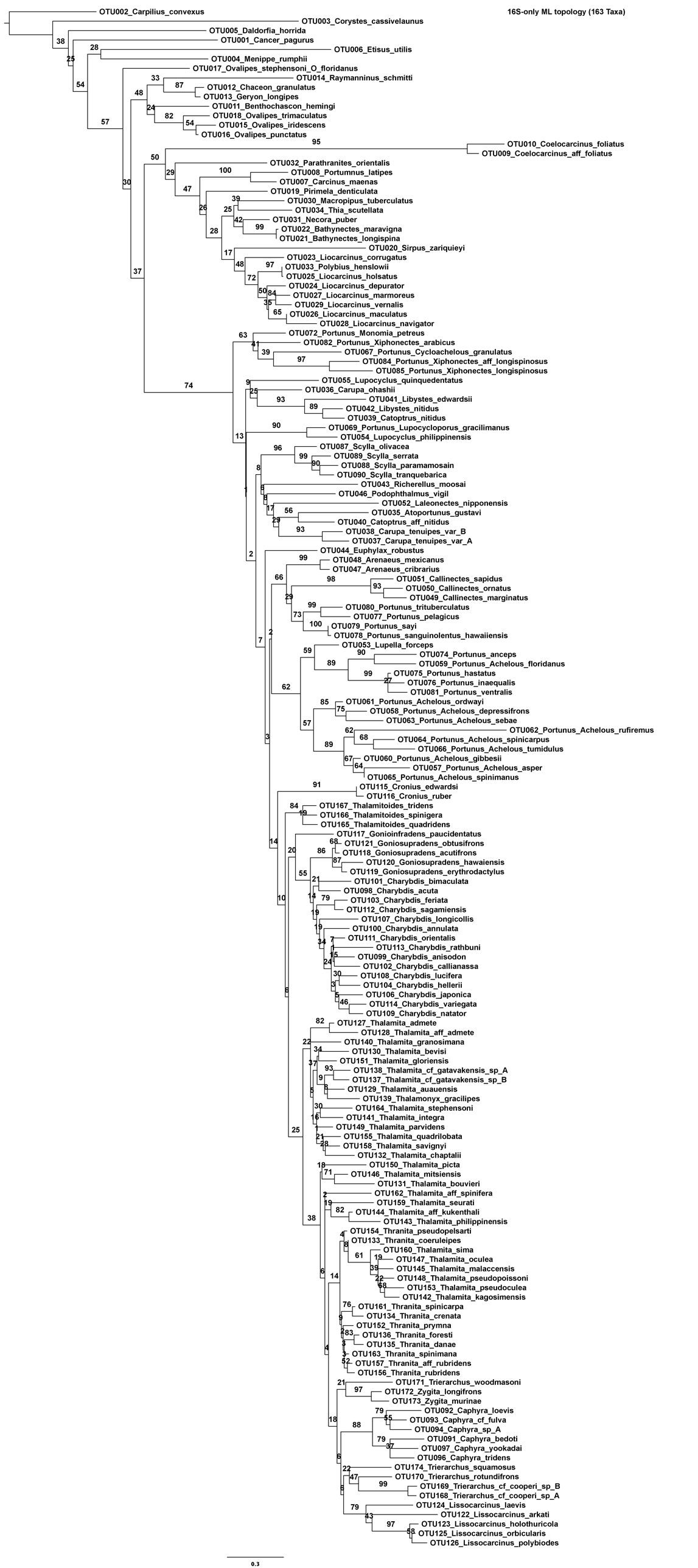

Supplement: Supplemental Information 2 — Support values (%) appear above each relevant node and are based on 500 bootstrap replicate ML searches. [file peerj-06-4260-s002.png]

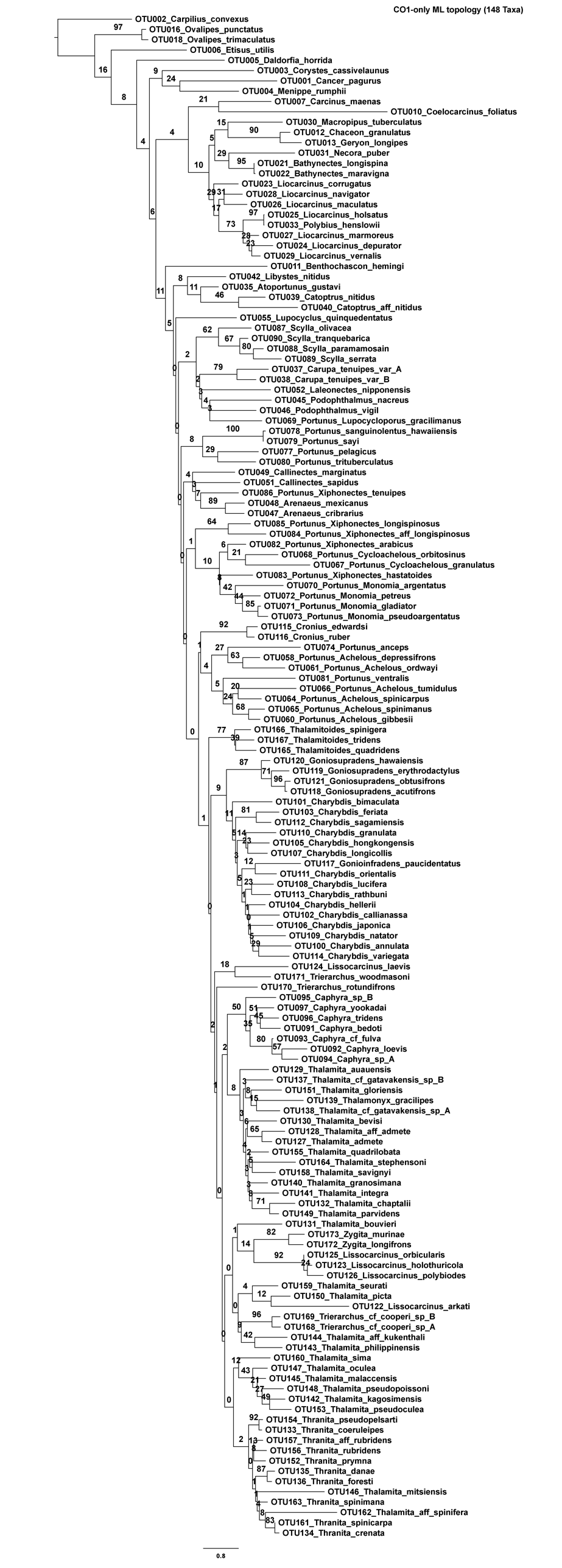

Supplement: Supplemental Information 3 — Support values (%) appear above each relevant node and are based on 500 bootstrap replicate ML searches. [file peerj-06-4260-s003.png]

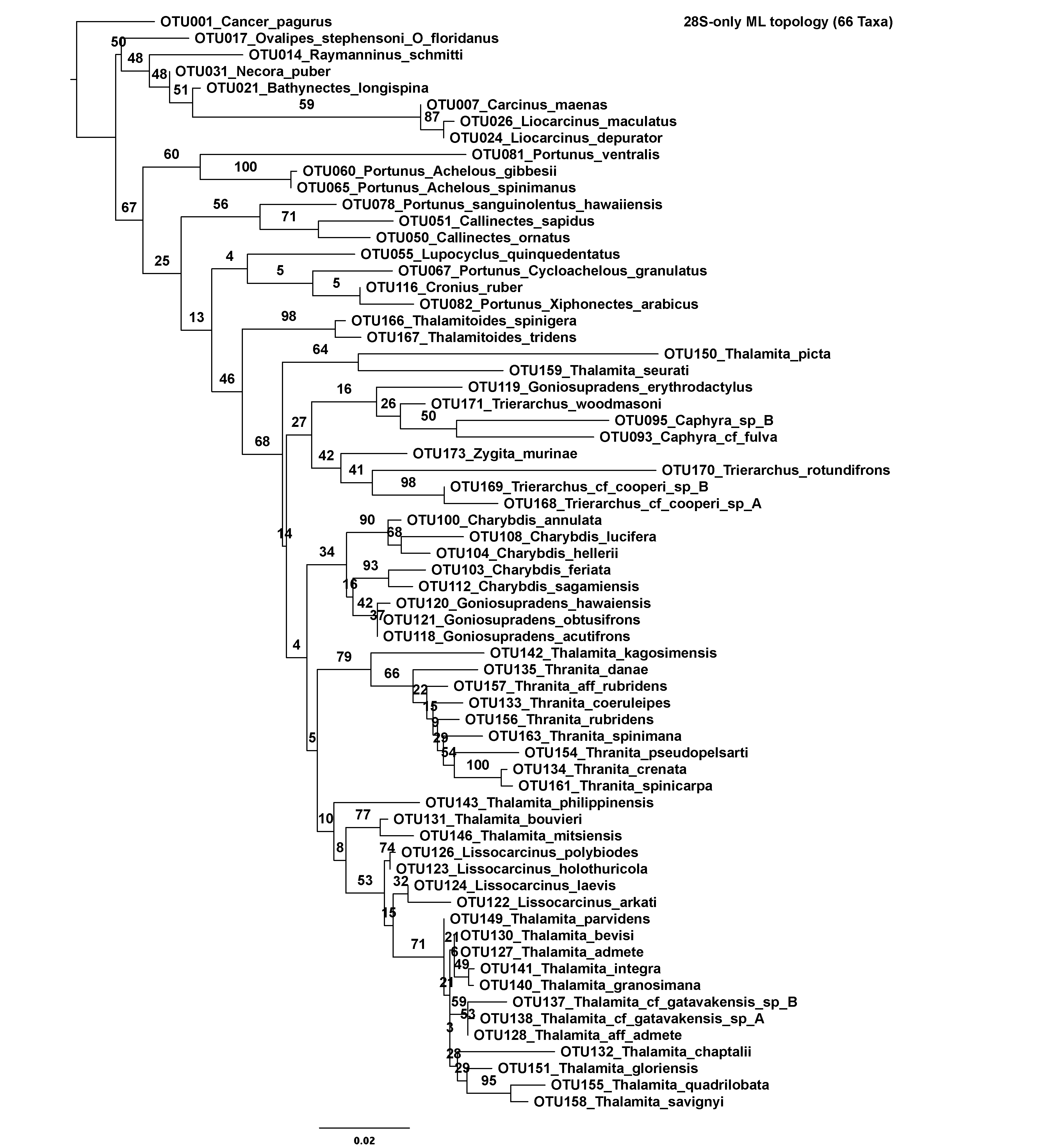

Supplement: Supplemental Information 4 — Support values (%) appear above each relevant node and are based on 500 bootstrap replicate ML searches. [file peerj-06-4260-s004.png]

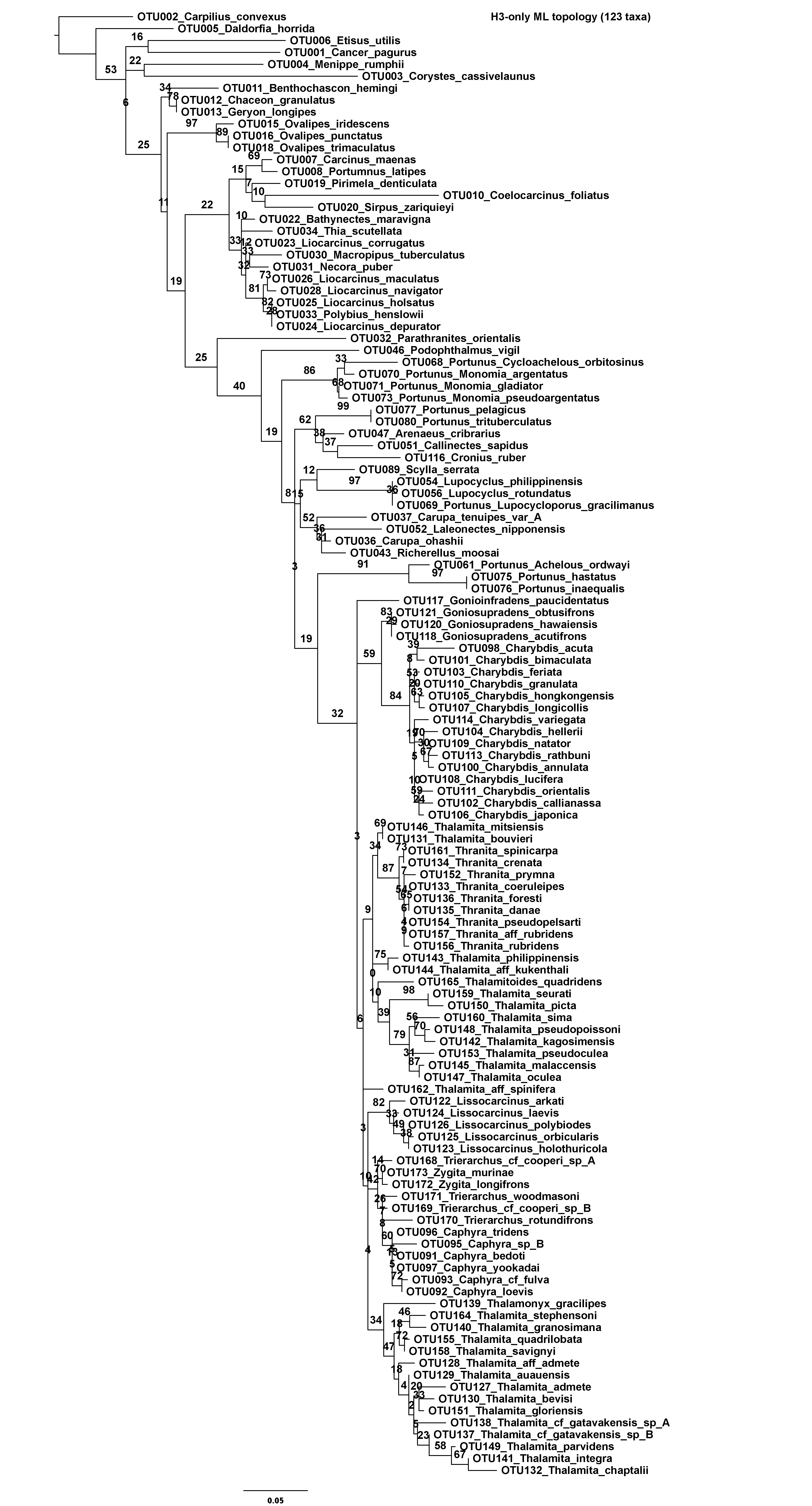

Supplement: Supplemental Information 5 — Support values (%) appear above each relevant node and are based on 500 bootstrap replicate ML searches. [file peerj-06-4260-s005.png]
