# Supplementary material for: Molecular phylogenetics of swimming crabs (Portunoidea Rafinesque, 1815) supports a revised family-level classification and suggests a single derived origin of symbiotic taxa
Source: PeerJ. 2018 Jan 23;6:e4260. doi: 10.7717/peerj.4260 (PMC5786103; doi:10.7717/peerj.4260)
Supplement: Supplemental Information 8 [file peerj-06-4260-s008.docx]

| **Marker** | **Taxa Count** | **Marker Subset** | **Alignment positions** | **Model for ML Runs** | **ML Partition ID** |
| --- | --- | --- | --- | --- | --- |
| 16S rRNA | 163 | 16S rRNA | 1-583 | TVM+I+G | 1 |
|  |  | tRNA-LEU | 584-653 | TVM+I+G | 1 |
|  |  | ND1 | 654-1105 | TrN+I+G | 2 |
|  |  |  |  |  |  |
| CO1 | 148 | Codon Pos. 1 | 1-657\3 | SYM+I+G | 1 |
|  |  | Codon Pos. 2 | 2-657\3 | F81+I+G | 2 |
|  |  | Codon Pos. 3 | 3-657\3 | GTR+G | 3 |
|  |  |  |  |  |  |
| 28S rRNA | 66 | D1 & D2 region | 1-1224 | GTR+I+G | 1 |
|  |  |  |  |  |  |
| H3 | 123 | Codon Pos. 1 | 2-327\3 | TrN+I | 2 |
|  |  | Codon Pos. 2 | 3-327\3 | JC+I | 3 |
|  |  | Codon Pos. 3 | 1-327\3 | GTR+G | 1 |
